# Family reported outcomes, an unmet need in the management of a patient's disease: appraisal of the literature

**DOI:** 10.1186/s12955-021-01819-4

**Published:** 2021-08-05

**Authors:** R. Shah, F. M. Ali, A. Y. Finlay, M. S. Salek

**Affiliations:** 1 Division of Infection and Immunity, School of Medicine, Cardiff University, Cardiff, UK; 2 School of Life and Medical Sciences, University of Hertfordshire, Hatfield, UK; 3 Institute of Medicines Development, Cardiff, UK

**Keywords:** Family member, Partner, Impact of illness, Quality of life, Family quality of life, FROM-16, Unmet need, Management of a patient's disease

## Abstract

**Background:**

A person’s chronic health condition or disability can have a huge impact on the quality of life (QoL) of the whole family, but this important impact is often ignored. This literature review aims to understand the impact of patients' disease on family members across all medical specialities, and appraise existing generic and disease-specific family quality of life (QoL) measures.

**Methods:**

The databases Medline, EMBASE, CINHAL, ASSIA, PsycINFO and Scopus were searched for original articles in English measuring the impact of health conditions on patients' family members/partner using a valid instrument.

**Results:**

Of 114 articles screened, 86 met the inclusion criteria. They explored the impact of a relative's disease on 14,661 family members, mostly 'parents' or 'mothers', using 50 different instruments across 18 specialities including neurology, oncology and dermatology, in 33 countries including the USA, China and Australia. These studies revealed a huge impact of patients' illness on family members. An appraisal of family QoL instruments identified 48 instruments, 42 disease/speciality specific and six generic measures. Five of the six generics are aimed at carers of children, people with disability or restricted to chronic disease. The only generic instrument that measures the impact of any condition on family members across all specialities is the Family Reported Outcome Measure (FROM-16). Although most instruments demonstrated good reliability and validity, only 11 reported responsiveness and only one reported the minimal clinically important difference.

**Conclusions:**

Family members' QoL is greatly impacted by a relative's condition. To support family members, there is a need for a generic tool that offers flexibility and brevity for use in clinical settings across all areas of medicine. FROM-16 could be the tool of choice, provided its robustness is demonstrated with further validation of its psychometric properties.

**Supplementary Information:**

The online version contains supplementary material available at 10.1186/s12955-021-01819-4.

## Background

A person’s chronic health condition or disability can have a huge impact on the quality of life (QoL) of the whole family. Sometimes this impact may be similar to or even greater than that experienced by the patient [1–3]. Although awareness of the impact of a person’s disease on family quality of life (FQoL) has recently been increasing, there is a need to measure this impact in the clinical setting to inform those providing support to the family. Turnbull et al. first proposed the term in 2000 and defined normal “family quality of life” as being "where the family's needs are met, and family members enjoy their life together as a family and have the chance to do things which are important to them" [4].

Golics et al.’s [5] detailed literature review of the impact of chronic disease on a patient's family revealed that various aspects of family life are affected by relative’s health condition. That review only identified information about a few disease areas and specialities [5] and concluded that there was no generic instrument at that time to measure disease impact on family members of patients.

The investigation of FQoL is a newly emerging field, with research now extending to many different areas of medicine. It is, therefore, timely to update the existing knowledge base on the family impact of disease and identify the development of new generic and disease-specific FQoL tools. This critical appraisal of the literature builds on the areas covered by Golics et al. [5] and summarises the greatly increased research activity over the last seven years. It aims to identify the impact of chronic disease on family members of patients across a range of medical specialities and appraise the characteristics and measurement properties of existing generic and disease-specific FQoL measures.

The definition of ‘family’ has changed over time and its use is no longer restricted to describing 'two parents and their children living under the same roof’. In this review, we use the term as defined by Poston et al. [6] as “People who think of themselves as part of the family, whether related by blood or marriage or not and who support and care for each other on a regular basis”. This review studies the impact of a patient's disease on all family members, including partners, whether or not they are also carers. Although the terms family caregivers, carers and informal caregivers are often used interchangeably, the only caregivers covered by this review are those unpaid carers (caregivers) who are family members or partners.

## Methods

### Search strategy

A search strategy was developed to identify studies published up to January 2020 that reported the impact of chronic disease on patients’ family members and partners. Six electronic databases were searched: Medline via OVIDSP; EMBASE via OVIDSP; CINHAL via EBSCO; ASSIA via ProQuest; PsycINFO Via OVIDSP; and Scopus using the PICO framework (Population: family members of chronic patients, Intervention: Patients chronic illness, Comparison: Non-applicable, Outcome: impact on family members) to identify and record the data (Additional file 1: Table S1a and S1b). The PICO framework was developed by the lead author and agreed by the other authors. The reference lists of included articles were also examined to ensure that all relevant articles were captured.

The search to identify existing generic and disease-specific FQoL measures was extended by combining search terms such as ‘family*or caregiver’ and ‘quality of life’ with the terms scale, index, measure, instrument, assessment, surveys, questionnaires, inventory, tools, generic or disease-specific (Additional file 1: Table S2). In addition, hand searches were carried out of the COnsensus-based Standards for the selection of health Measurement Instruments (COSMIN) [7] database and the reference lists of relevant articles. Google Scholar was searched for articles reporting development or psychometric properties of the instruments identified.

### Eligibility criteria

Articles were included in the review if the source was an original paper, in the English language and measuring the impact of chronic illness or disability on patients' family members/partner using a valid tool. Studies were excluded if they were book chapters, congress abstracts, if they used qualitative methodology or if the caregiver was not a family member. This review paper is in two parts, the first part focuses on the impact of a patient’s disease on family members and the second part appraises the instruments available to measure this impact. As one of the inclusion criteria for the second part was only to include quantitative techniques, it was felt methodologically appropriate to align the two parts by including only quantitative studies in the first part. We recognize this could be considered as a limitation of the study.

### Screening

In the first stage of article screening, duplicates were removed, and irrelevant titles and abstracts were discarded based on eligibility criteria. In the second stage, full-text articles of potentially relevant abstracts were read and assessed against eligibility criteria by RS to make a final decision about study selection agreed by MSS and AYF.

### Data extraction

Data extraction was carried out by RS and was discussed using an iterative process with other members of the research team (MSS and AYF). The data extracted included authors, publication year, country of study, study design, sample size, patients’ chronic disease, family member gender, relationship to the patient, impact on the family members and tools used to measure this impact (Additional file 1: Table S3).

A separate data extraction table was used for recording psychometric properties of identified family QoL instruments.

### Synthesis of data

We used a thematic approach to synthesise findings. Selected papers were carefully read by RS: in case of ambiguity, papers were discussed with FMA, AYF and MSS to ensure accuracy of data extraction. The data on the impact of patients’ disease on family members were summarised as short notes for the 86 studies. These notes were then coded to capture their essence and finally, codes were sorted into potential themes.

### Quality assessment and risk of bias

The quality of selected papers and assessment of risk of bias was evaluated using the Joanna Briggs quality assessment tool for cross-sectional and cohort studies, with the involvement of MSS and AYF [8]. The checklist consists of 8–11 questions with answers “yes”, “no” and “unclear”. When all answers were “yes”, the study was considered to have less chance of bias and if any answer was “no”’ the study was classified as having a risk of bias. The PRISMA principles were followed to ensure robustness of the review as well as minimising bias [9].

## Results

### Screening

**Fig. 1:**
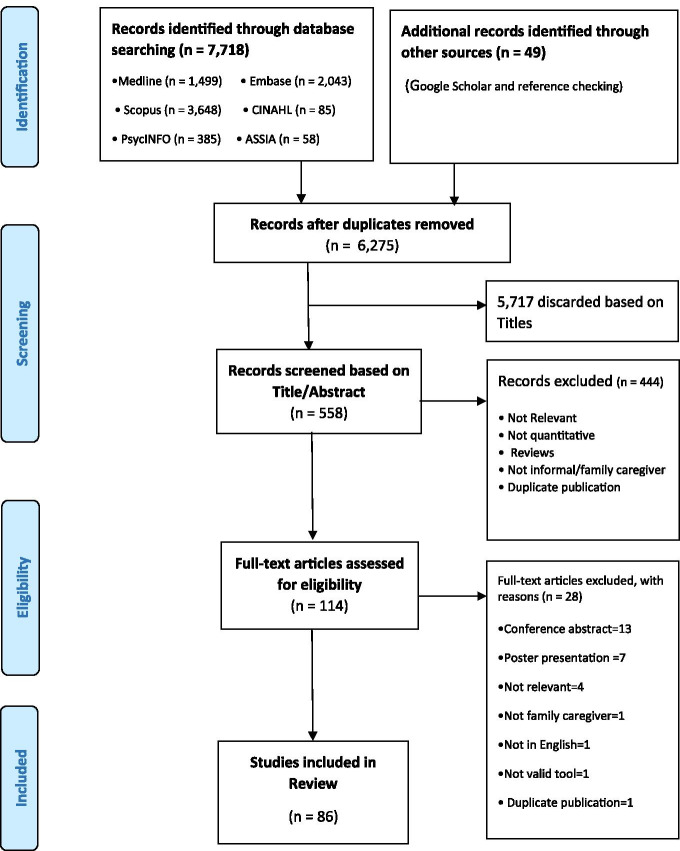
PRISMA flow diagram of article selection

A total of 7,767 articles were identified. After removing duplicates and irrelevant titles, 558 abstracts were screened. The resultant 114 articles underwent full-text review, 86 articles met all inclusion criteria and were included in the final analysis (Fig. 1).

### Study characteristics

**Fig. 2:**
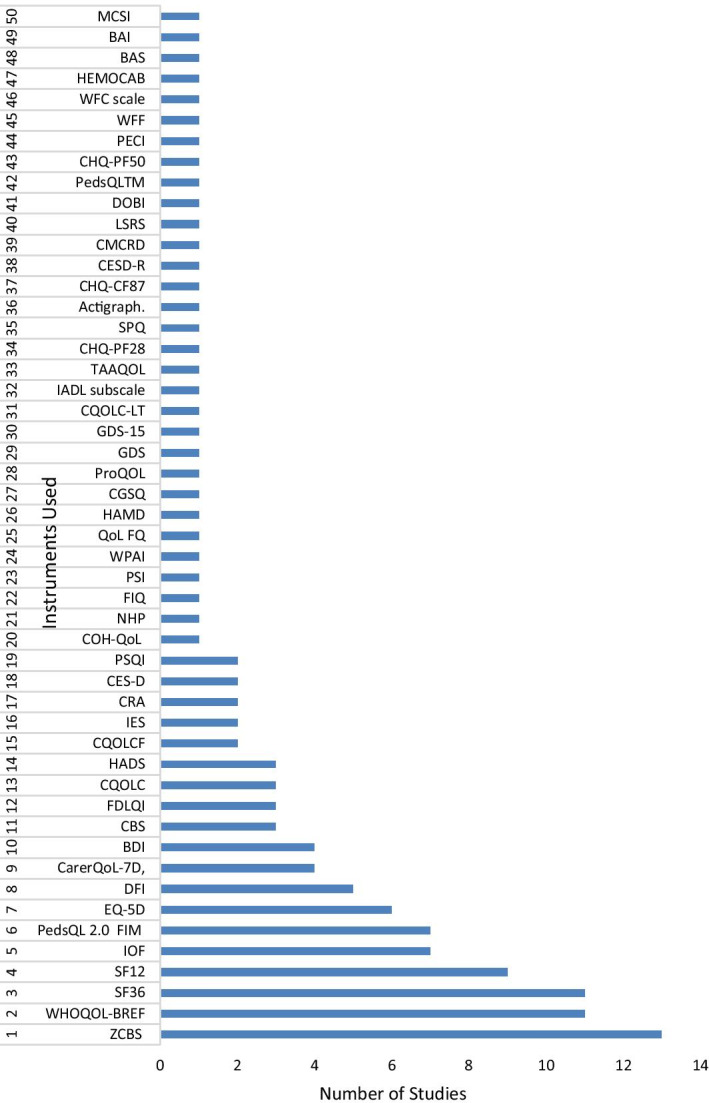
Instruments used in the reviewed studies to measure the impact of the disease on family members/partners. **ZCBS**: Zarit Caregiver Burden Scale; **WHOQOL**: The World Health Organization Quality of Life; **SF36**: The Short Form (36) Health Survey; **SF12**: 12-item Short Form Health Survey; **IOF**: Impact on Family Scale; **EQ-5D**: Euroqol- 5 Dimension; **PedsQL 2.0 FIM**: PedsQL TM 2.0 Family Impact Module; **DFI**: Dermatitis Family Impact questionnaire; **CBS**: Caregiver Burden Scale; **CarerQoL-7D**: Care-related Quality of Life instrument-7 Dimension; **BDI**: Beck Depression Inventory; **FDLQI**: Family Dermatology Life Quality Index; **CQOLC**: Caregiver Quality of Life Index-Cancer; **HADS**: Hospital Anxiety and Depression Scale; **CQOLCF**: Caregiver Quality of Life Cystic Fibrosis; **IES**: The Impact of Event Scale; **CRA**: The Caregivers Reaction Assessment Scale; **CES-D**: Centre for Epidemiologic Studies Depression Scale; **COH-QOL**: City of Hope Quality of life Questionnaire: **NHP**: The Nottingham Health profile questionnaire; **FIQ**: Family Impact Questionnaire; **PSQI**: Pittsburgh Sleep Quality Index; **PSI**: The Parenting Stress Index Questionnaire; **WPAI-SHP**: The Work Productivity and Activity Impairment-Specific Health Problem V2.0; **QoLFQ**: QoL Family Questionnaire; **HAMD**: Hamilton Depression Scale; **CGSQ**: the Caregiver Strain Questionnaire. **ProQOL**: Professional Quality of Life; **GDS**: Geriatric Depression Scale; **GDS-15**: Geriatric Depression Scale-15; **CQOLC-LT**: Caregiver Quality of life index-Liver Transplantation; **IADL subscale**: Instrumental Activities of Daily Living; **TAAQOL**: TNO-AZL Questionnaire for Adult Health-Related Quality of life; **CHQ-PF28**: Child Health Questionnaire-Parent Form-28; **SPQ**: Sibling Perception Questionnaire; **CHQ-CF87**: Child Health Questionnaire-Child Form 87; **CESD-R**: Centre for Epidemiologic Studies Depression Scale (revised); **CMCRD**: Caring for my Child with a Juvenile Rheumatic Disease; **LSRS**: Lifespan Sibling Relationship scale; **DOBI**: Dutch Objective Burden Inventory; **CHQ-PF50**: Child Health Questionnaire-Parent Form 50; **PECI**: Parent Experience of Child Illness; **WFF**: Work-Family Facilitation scale; **WFC scale**: Work-Family Conflict scale; **PedsQLTM**: Pediatric Quality of Life Inventory TM; **HEMOCAB**: Hemophilia Associated Caregiver Burden Scale; **BAS**: Burden assessment Scale; **BAI**: Becks Anxiety Inventory; **MCSI**: Modified version of Caregiver Strain Index

**Fig. 3:**
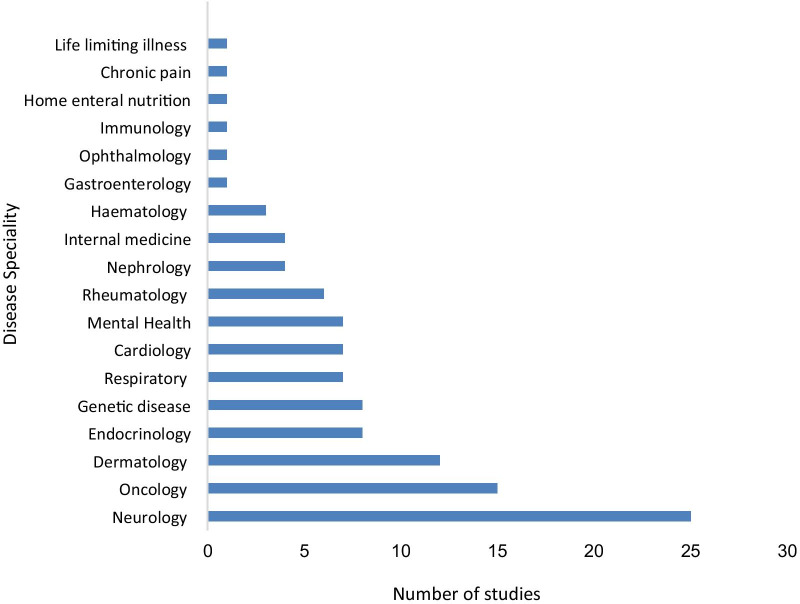
Disease speciality and number of studies included in this review

Eighty-one studies were cross-sectional, and five studies were longitudinal prospective cohort studies with follow-up ranging from one month to two years. The studies explored the impact of a relative's disease on a total of 14,661 family members, mostly 'parents' or 'mothers', using 50 different tools across 18 specialities including neurology, oncology and dermatology and covering 33 countries including the USA, China, and Australia (Figs. 2 and 3; Additional file 1: Table S4 and S5). The most widely used tool to measure the impact of a patient's disease on a family member was the Zarit Caregiver Burden Scale (13 studies) followed by WHOQOL (11), SF-36 (11), SF-12 (nine), IOF (seven) and EQ-5D (six) (Fig. 2). While most of the articles reported the impact of a single chronic disease on family members, ten studies included more than one chronic condition, allowing comparison of the family impact of different diseases.

### Quality assessment and risk of bias

Thirteen cross-sectional studies and one cohort study did not mention confounders and strategies to address them while one cohort study did not mention reasons for loss of follow-ups. However, the remaining requirements were met for all of these studies, which all fulfilled the minimum criteria for quality. None of the 86 studies was rejected based on their quality or risk of bias. Overall, all studies were moderate to high quality (Additional file 1: Table S6).

### Synthesis of findings—key impact areas

This review revealed a huge impact of patients’ illness on family members’ QoL [10–18]. In general, relatives’ chronic diseases impacted family members in similar ways, with some conditions such as cancer having a bigger impact than others. Some common themes identified in this review are discussed below.

## Emotional and psychological impact

Caring for a relative’s chronic disease affects family members’ lives in many ways, impacting their emotional and psychological wellbeing [19]. The family members caring for their relative with a chronic disease were at risk of themselves developing a mental health condition, with an adult offspring or spouse at higher risk than other family members [10, 20, 21], and suffered similar psychological distress, depression and anxiety levels to that of the patient [22–24]. The presence of anxiety and/or depression in the family member was the most consistent factor influencing family members’ burden and perceived health-related QoL (HRQoL) [25, 26].

### Nature of relationship and psychological impact

Mothers of children with chronic disease experienced high rates of stress, anxiety and depression [15, 27–30]. Parenting stress was higher when a child was of pre-school age [31, 32] and displaying disruptive behaviours and developmental disabilities [33] or showing flares due to increased severity of their condition [34, 35]. Some parents perceived the increased caring demands of a sick child as 'intrusive' which led to higher levels of parental stress and psychological distress [36] affecting the perception of burden experienced by the mother [37]. However, this emotional distress did not result in mothers being less caring of the sick child [38]. The children of mothers with a chronic condition experienced more symptoms of hyperactivity and inattention, especially when the mothers had psychological problems [39]. Siblings of children with a more severe chronic condition and an unpredictable prognosis reported more internalising of problems and behavioural difficulties than siblings of children with a chronic condition that followed a daily routine treatment pattern [40]. However, poor emotional health of siblings of children with controlled asthma was not related to disease severity [41]. Moreover, what is worrying is that parents are sometimes unaware of the impact of their child’s disease on their other children [42].

### Gender differences

Female family members, spouses and mothers, experienced significantly higher rates of depression and anxiety than male family members [15, 21, 25, 28, 29, 43] and the impact was greater when patients suffered from a severe disease such as a long-term mental health condition [44]. However, two studies showed fathers experiencing more stress [45] and lower HRQoL [46] than mothers. Such paternal outcomes could be explained based on increased stressors arising from disease flares, such as additional medical visits and medical bills, both of which could be particularly distressing for fathers compared to mothers [46]. The reverse gender difference was found in siblings of a patient, with female siblings experiencing a lower QoL than male siblings [40].

## Impact on physical health

Caring for a relative with a chronic disease can have an impact on family members’ physical health owing to the burden resulting from the relative’s functional disabilities, cognitive impairment [27, 47, 48], medication management [49] duration of care [43, 50] and total daily hours spent on assisting patients with basic activities of daily living and medical tasks [12, 50–52]. Caring for their relative can leave family members overwhelmed and physically exhausted [53, 54], which may result in compassion fatigue. It is not the total number of years of caregiving that contributed to differences in compassion fatigue, but the number of hours per week [55], suggesting that intensity of caring rather than duration is the critical factor. Furthermore, family members of people with less severe chronic diseases reported only a moderate burden on QoL [56, 57], indicating that caregiving burden is related to the severity of the patient's disease and the family member's perception of burden [35, 58].

### Sleep

The physical health of family members caring for their relative was impacted by poor sleep quality [59–64]. Meltzer et al. [61] found that parents of ventilator-assisted children experienced shorter sleep duration and greater variability in sleep quality impacting their physical health compared to parents of healthy children. In the mothers of children with Duchenne muscular dystrophy, impaired sleep quality was related to the disease duration [62], while the sleep disturbance in the parents of children with atopic disease was related to the children’s sleep disruption [63]. The partners of cancer patients experienced poor sleep quality: there was a significant correlation between patients' and their partners' sleep quality and sleep onset latency [60]. Although partners used medication to minimise the negative impact of sleep problems, Chen et al. [60] argue that this could have affected their ability to respond to the needs of the patient, indicating that many family members may be hesitant to use drugs to aid sleep.

## Impact on social, leisure and daily activities

Family members caring for a relative with a chronic condition experience a considerable impact on their social, leisure and daily activities [38, 51, 58, 65, 66], with women reporting greater disruption than men [67]. Most family members caring for their relative reported difficulties in combining caring tasks with daily activities [29, 68, 69].

Parents of children with chronic disease reported less opportunity for leisure and social activities [38, 53, 68, 70]. The high caregiving demands of children with developmental disabilities, especially if outwardly visible, contributed to social isolation [33]. The parents of children with obsessive compulsive disorder experienced interruptions in social life such as postponing social activities [71]. Parents of children receiving palliative care felt little desire to go out, indicating that the severity of their child's disease led to a loss of interest in leisure activities [72].

There seems to be a cultural aspect to the impact of caregiving on social life. Japanese caregivers reported high social scores on the Zarit burden scale [73], even when their perception of general health was lower than that of the care recipient. This indicates that unlike Western caregivers, Japanese caregivers do not report their feelings about their social life being impacted by caregiving [73]. Arab mothers of children with disabilities experienced reduced social interactions and lower QoL due to the cultural beliefs and the stigma attached to having a child with a disability [48].

## Impact on family relationships

A relative's chronic condition has an impact on the relationships among family members and between the patient and the family members [29, 74, 75]. Caring for a family member not only impacts the carer but also the whole family [16, 76] and better family relationships improved QoL for both patient and family members [35, 69, 77].

Mothers caring for children with attention deficit hyperactivity disorder and oppositional developmental disorder (ODD) experienced negative feelings towards their affected child. Some mothers attributed their child’s ODD to increased conflicts between them and their partners [74]. However, having more children was seen as being protective against partner conflict and maternal hostility, as siblings could assist the mother by caring for the sick child, thereby reducing parental stress and negative feelings towards the child [74]. Conversely, siblings may internalise their emotional reactions to the situation, leading to behavioural problems [40]. Better alignment and coordination between parents and involving the siblings, however, could lead to family cohesion, tackling the problem together.

Partners of patients experienced poor sexual life and relationship quality because of the patient's symptoms [68, 78], with a significant decrease in the partners’ ability to spend quality time with the patient [70], leading to marital conflicts [68]. For many, the caregiving role restricted them from having more children [72]. Knap et al. [72] reported that 48% of parents of children with life-limiting illnesses choose not to have more children because of their child's illness and associated caring responsibilities.

## Financial impact

Caring for a relative with a chronic disease can necessitate increased expenditure [15, 31, 67, 68, 79–83]. In an Australian study, the annual personal cost for mild, moderate, and severe atopic dermatitis was calculated at Aus$330, 818, and 1255, respectively, with most being spent on medication, dressings and non-irritant clothing [64]. In a Swedish study, 20% of parents reported experiencing financial difficulties even after the cost of the chronic disease treatment was covered by the welfare system [84]. The family members reduced their working hours or left their jobs to take up their caring responsibilities. This and the expense of hospital visits contributed to their financial difficulties [64, 84, 85].

## Impact on work

Work was seen to have a positive impact on the QoL of mothers, as it provided temporary relief from their caring role, time to socialise and offset the financial burden [47, 71]. However, many family members caring for their relative suffered work impairment [75, 86] and had to give up their jobs, change jobs, alter career choices or reduce their work hours to look after an ill family member and to manage hospital visits [64, 70, 87, 88].

## Positive aspect of caregiving

Despite the physical, social and psychological impact that a relative having a disease has on family members, many family members reported a positive experience of caregiving, with older family members reporting more satisfaction than younger ones [55]. Meriggi et al. [67] reported 93.5% family members caring for their relative were happy with their role. Son et al. [77] attribute positivity in family members caring for cancer patients to their spiritual upliftment. Awadalla et al. [89] attribute this positive impact to family cohesion, and an attitude of hopefulness. Adult siblings caring for their parents reported that they see caregiving as a way of giving something back to parents [90]. Although the health status of family members with caring experience was lower than that of non-carers in an Australian study, the difference in scores did not reach the minimal important difference (MID) magnitude for either the mental or physical domains of SF-12, suggesting that caregivers might be satisfied in their caring roles [91].

### Existing family QoL instruments

The appraisal of the family QoL measures identified 48 instruments measuring the impact of a patient's disease on family members. Forty-two of the instruments are disease or speciality specific and are limited to that particular group of patients. The properties of these measures are summarised in Tables 1 and 2.

**Table 1:** Summary characteristics of Family quality of life Measures—disease/speciality specific

Name of measure/key references	Country	Disease/speciality	Population	Language/translation	Completion time	Origin	Domains	Number of items	Scale	Mode of administration
1. Family Dermatology Life Quality Index (FDLQI) Basra et al. [92]; Basra et al. [93]	UK	Speciality-specific (Dermatology)	Family members of patients with skin disease	English/Italian Persian and Ukrainian	2–3 min	Semi-structured interviews with family members or partners of patients with a variety of skin diseases	Emotional and physical wellbeing, relationships, social life, leisure activities, burden of care, impact on job study, housework and expenditure	10	4-point Likert	Self-report
2. Dermatitis Family Index (DFI) Lawson et al. [94]; Beattie &, Lewis-Jones [95]	UK	Disease-specific (Dermatitis)	Parents and other family members of children with Atopic Dermatitis	English/Arabic, Chinese, Czech, Dutch, French, Greek, Italian, Japanese, Norwegian, Polish, Portuguese, Spanish, Swedish, Ukrainian	2–3 min	Qualitative interviews with family members/focus group	Housework, food preparation and feeding, sleep, family leisure activities, time spent on shopping for the family, expenditure, tiredness, emotional distress, relationships between the main carer and partner or between the main carer and other children and helping with treatment	10	4-point Likert	Self-report
3. Parents’ Index QoL Atopic Dermatitis (PiQoL) McKenna et al. [96]; Meads et al. [97]	UK, Netherlands, Germany, Italy, Spain, France, USA, Switzerland	Disease-specific (Atopic Dermatitis)	Caregiver of children with Atopic Dermatitis, aged 8 years or younger	English/Dutch, Italian, French, German, Spanish	4–5 min	Qualitative interviews with parents of children with Atopic dermatitis in the UK, Netherlands and Italy	Needs that can be influenced by a child having atopic dermatitis (e.g., need for child to have a safe and successful future, need for rest and relaxation, need for Self-respect, need for independence)	28	Dichotomous	Self-report
4. QoL in primary caregivers of children with atopic dermatitis (QPCAD) Kondo-Endo et al. [98]; Katsunuma et al. [99]	Japan	Disease-specific (Atopic Dermatitis)	Primary caregivers of children with atopic dermatitis	English	1–2 min	Semi-structured interviews	Four domains-Exhaustion, worry about atopic dermatitis, family cooperation, and achievement	19	5-point Likert	Self-report, mail
5. Childhood Atopic Dermatitis Impact Scale (CADIS) Chamlin et al. [100] Chamlin et al. [101]	USA	Disease-specific (Atopic Dermatitis)	Children with Atopic dermatitis younger than six years and their families	English	6 min	Focus groups with parents and expert & Lit review	Five domains, three of whom refer to the impact on the family: family and social function, sleep, and emotions	45	5-point Likert	Self-report
6. Psoriasis Family Index (PFI) Eghlileb et al. [102]; Basra et al. [103]	UK	Disease-specific (Psoriasis)	Family members of psoriasis patients	English	2–3 min	Interviews with relatives of people with psoriasis	Frustration, worry about the reaction of other people, worry about their future, relationships, housework due to psoriasis and to treatment, time spent on treatment, social life, sporting activities, leisure activities, type of clothes, routine shopping and sleep	14	4-point Likert	Self-report
7. Atopic dermatitis Burden Scale (ABS)Méni et al. [104]	France	Disease- specific (Dermatology)	Parents of children with Atopic dermatitis (AD)	French, English US, German, Italian, Spanish, Danish, Romanian and Georgian	NF	Literature review; educational workshop/discussion groups with parents of children with AD; feedback from expert HCPs/Parent association AD	Four domains-Family life, budget & work, daily life and treatment	14	4-point Likert	Self -report
8. Haemangioma Family Burden (HFB) questionnaire Boccara et al. [105]	France	Disease- specific (Dermatology)	Parents of children with Infantile haemangioma(IH)	French/US and UK English, Spanish, Italian and German	NF	Literature review, interviews with healthcare professionals (paediatricians, dermatologists, nurses) and with the parents of children that have or have had IH of varying severity	Five domains-Family life, relationship and work, emotions/feelings, psychological and disease management	20	3-point Likert	Self-report
9. FamilyPso Mrowietz et al. [106]	Germany	Disease-specific	Partners or family of psoriasis patient	English	NF	Literature reviews and interviews with relatives of people with psoriasis	Four domains -Emotional impact of the disease, impact on daily activities and work or school and treatment characteristic, and influence on leisure activities and personal relationships	15	5-point Likert	Self-report
10. Epidermolysis Bullosa Burden of Disease (EBBoD) Dufresne et al. [107]	France	Disease-specific (Epidermolysis Bullosa)	Families of children with epidermolysis bullosa (EB)	French	NF	Verbatim report based literature review and data collection from parents of patients during a one‐to‐one session with the same social worker	Four domains-Family life , child’s life , disease and treatment , and economic and social impact	20	7-point Likert	Self-report
11. Family Burden Ichthyosis (FBI) Dufresne et al. [108]	France	Disease-specific (Ichthyosis)	Families of children with Ichthyosis	French	NF	Literature reviews and interviews with patients, parents and experts	Five domains- Economic, daily life, , familial and personal relationship, work and psychological impact	25	4-point Likert	Self-report
12. Family burden of Incontentia pigmenti (IP) F’BoIP questionnaire [109]	France	Disease- specific (Dermatology)	Parents/family members of children with IP condition	French/US English	NF	Interviews with dermatologists, patient-reported outcome (PRO) experts and IP parents	Four domains -Social life and family life, Professional life and renunciation, Daily life and Economic impact	20	6-point Likert	Self-report
13. Parents’diabetes QoL Questionnaire (PDQoL) Vandagriff et al. [110]; Faulkner et al. [111]	USA	Disease-specific (Diabete Type 1)	Parents of children with type 1 diabetes	English	NF	NF	Three domains- Life satisfaction, impact of disease, and worries related to the disease	42	5-point Likert	Self-report
14. Well-being and Satisfaction of CAREgivers of children with Diabetes Questionnaire (WE-CARE) Cappelleri et al. [112]	USA	Disease-specific (Diabetes Type 1)	Primary caregivers and parents of children with Diabetes type 1	English/Portuguese/ Spanish/Swedish	10–15 min	Interviews with children and caregivers/paediatricians	Four domains- Psychosocial well-being, ease of Insulin use, treatment satisfaction, and acceptance of Insulin administrations	37	5-point Likert	Self-report
15. Diabetes family impact scale (DFI-S) Katz et al. [113]	USA	Disease-specific (Diabetes Type 1)	Parents of children and adolescents with type 1 diabetes	English	NF	Interview with parents of children with diabetes and multidisciplinary expert panel	Four domains- School, work, finances and family well-being	14	4-point Likert	Self-report
16. Parent Ear Nose and Throat QoL questionnaire (PAR-ENT-QoL) Berdeaux et al. [114]	France, Italy, Germany Czech republic, Portugal	Speciality-specific (Ear-nose-throat infection/pharyngitis)	Parents of children with ENT infections	France, Italy, Germany, Czech, Portugal	5 min	Interviews with families	Three domains- an emotional score, a daily disturbance score, and a global score	14	5-point Likert	mail
17. Food Allergy Quality of Life Parent Burden (FAQLQ-PB) Cohen et al. [115]	USA	Disease-specific (Food Allergy)	Parents of children with Food allergy	English/Chinese	5–7 min	Interviews/focus groups with caregivers	Three domains- Issues concerning going on vacation, social activities and worries and anxieties over the previous week	17	7-point Likert	mail
18. Caregiver Quality of Life Cystic Fibrosis (CQOLCF) Boling et al. [116]	USA	Disease-specific (Cystic Fibrosis)	Caregivers Patients with Cystic Fibrosis	English	7–8 min	Expert review/care staff team	Four domains-The physical well-being, emotional well-being, social/family well-being, and functional well-being	35	5-point Likert	Telephone
19. OverActive Bladder Family Impact Measure OAB-FIM Coyne et al. [117]	USA	Disease-specific (Overactive Gall Bladder)	Family members of a patient with Overactive bladder	English Spanish Turkish	NF	Focus group with Family members of patients with Overactive bladder	Six domains- (Irritation, activities, travel, concern) for all family members and sleep, sex for spouses and significant others	19	5-point Likert	Self -report
20. ITP- Idiopathic thrombocytopenic purpura— Parental Burden QoL questionnaire (ITP—PB) Barnard et al. [118]	Canada, USA	Speciality specific (Hematologic disorder)	Parents of children with a hematologic disorder	English	5–7 min	Interview with parents/health professionals	Six domains: concerns related to diagnosis/investigation, treatment/disease monitoring, monitoring of child's activities, interference with daily life, disease outcome, and emotional impacts	26	5-point Likert	Self-report
21. Huntington’s disease quality-of-life battery for carers (HDQoL-C) Aubeeluck & Buchanan [119]	UK	Disease-specific (Huntington’s disease)	Family caregivers of persons with Huntington’s Disease	English	21 min	Qualitative interview/Photovoice	Four domains- Demographic and objective information; practical aspects of caregiving; satisfaction with life; feelings about living with Huntington’s	34	11-point Likert	Self-report
22. Huntington’s disease quality-of-life battery for carers short form (HDQoL-C-SF) Aubeeluck et al. [120]	France, Italy	Disease-specific (Huntington’s disease)	Family caregivers of persons with Huntington’s Disease	English/French, Italian, German, Polish, Portuguese, Spanish and Swedish,	NF	312 carers from France and Italy completed HDQoL-C to develop a shortened version of the HDQoL-C	Two domains-Satisfaction with life; feelings about living with Huntington’s disease	20	11-point Likert	Self-report
23. Alzheimer’s Carers Quality of Life Instrument (ACQLI) Doward [121]	UK France Germany, Italy, Spain	Disease-specific (Alzheimer’s)	Carers of patients with Alzheimer’s disease	English	NF	NF	The single domain of carer QoL	30	Dichotomous (true/not true)	Self-report
24. Care related Quality of care—Multiple Sclerosis (CAREQOL-MS) Benito-Leon et al. [122]	Spain	Disease-specific (Multiple Sclerosis)	Caregivers of Multiple Sclerosis	English/Spanish	NF	Focus groups were organized with MS patients and caregivers. /MS expert	Five domains-Physical burden and global health; social impact; emotional impact; need of support; emotional reactions to patient’s psychic status	24	5-point Likert	Self-report
25. Parkinson Disease Questionnaire for Carers (PDQ-Carer) Jenkinson et al. [123]	UK	Disease-specific (Parkinson Disease)	PD carers	English	NF	Carer Survey s registered with local branches of Parkinson's UK	Four domains- Social and personal activities; anxiety and depression; self-care; stress	29	5-point Likert	Self-report
26. Parkinson Disease Questionnaire for Carers Summary Index (PDQ-Carer-SI) Morley et al. [124]	UK	Disease-specific (Parkinson Disease)	PD carers	English	NF	Carer Surveys registered with local branches of Parkinson's UK	Single summary index score computed using the four subscales of the PDQ-Carer	29	5-point Likert	Self-report
27. Parkinsonism Carers QoL (PQoL Carers) Pillas et al. [125]	UK	Disease-specific (Atypical Parkinsonism)	Relatives/partner of patients with atypical Parkinsonism (AP)	English	NF	Qualitative interviews with relatives/partner of a person with AP and Consultation with AP experts	Single domain of carer QOL	26	5-point Likert	Self-report
28. Family Outcome Measure -40 (FOM-40) Migliorini et al. [126]	Australia, New Zealand, Canada, UK	Disease-specific (Traumatic brain injury)	Families with relative having a traumatic brain injury	English	NF	Social workers from 12 rehabilitation centres across Australia, New Zealand, Canada, and the UK	Seven domains-Family member coping, family cohesion, support demands (burden), relative adjustment, adequacy of service, family member resilience, sustainability of family support	40	4-point Likert	Self-report
29. Caregiver Quality of life (CGQOL) Vickrey et al. [127]	USA	Disease-specific (Dementia)	Family caregivers of people with Dementia	English	17 min	Interviews with carers of Dementia Patients	Ten Domains; Assistance with instrumental activities of daily living; assistance with activities of daily living; role limitations due to caregiving; personal time; family interaction; demands of caregiving; worry; spirituality and faith; benefits of caregiving; caregiver feelings	80	3-point and 5-point Likert	Telephone interview
30. Caregiver Dementia Quality of Life (C-DEMQOL) Brown et al. [128]	UK	Disease-specific (Dementia)	Family members of people with Dementia	English	15 min	Literature reviews/qualitative interviews with family carers and support staff, /Focus groups with carers and staff	Five domains- Responsibilities and personal needs; wellbeing; carer role and relationships with the person with dementia; feelings about future and carer support	30	5-point Likert	researcher administered/Self-report
31. Family Impact Scale-Oro-facial (FIS—OFD) Locker et al. [129]	Canada	Disease-specific (Oro-facial Disorder)	Parents of children with Oro‐facial conditions	English	5 min	Review of existing child health status and family impact questionnaires, interviews with 41 parents/caregivers	Four domains-Parental and family activity, parental emotions family conflict and financial burden	14	5-point Likert	Self-report
32. Quality of Life in life-Threatening Illness–Family Carer Version (QoLLTI–F) Cohen et al. [130]	Canada	Speciality specific (Oncology)	Caregivers of cancer patients receiving palliative care	English/French	< 10 min	Previous research and expert review	Seven domains-Carer’s own state, relationships, carer outlook, quality of care, patient condition, finances, environment	16	11-point Likert	Self-report
33. CareGiver Oncology Quality of Life questionnaire (CarGOQoL) Minaya et al. [131]	USA	Speciality specific (Oncology)	Caregivers of cancer patients	English/French	6 min	Qualitative interviews with informal caregivers of cancer patients	Ten domains-Psychological wellbeing, burden, relationship with healthcare, administration and finances, coping, physical well-being, Self-esteem, leisure time, social support and private life	29	5-point Likert	Self-report
34. Caregiver Quality of Life Index–Cancer Weitzner et al. [132]	USA	Oncology Speciality- specific	Primary caregiver of cancer patients	English, Turkish, Korean, Chinese	10 min	A semi-structured interview with family caregivers, physicians, nurses and social/Expert Review	Four domains-Burden, disruptiveness, positive adaptation, and financial concern	35	5-point Likert	Self- report
35. City of Hope QoL Scale–Family Version Ferrell et al. [133] City of Hope. [134]	USA	Speciality-specific (Oncology)	Family caregivers of cancer patients	English and Spanish	NF	In-depth qualitative interviews with cancer survivors over five years Pilot	Four domains-Physical, psychological, social, spiritual	37	11-point Likert	Self- report, mail
36. Caregiver Impact Questionnaire (CIQ Survey Otitis media) Boruk et al. [135]	USA	Disease-specific (Acute Otitis Media)	Parents of children with acute otitis media	English	NF	Previous research/Expert Panel/parents/non-medical volunteer	Four domains- Caregiver physical Functional health status (FHS), caregiver emotional FHS, & caregiver QoL rating and sibling impact score	10	Mix of 7 and 5-point Likert and visual-analog scale	Self- report
37. Acute Otitis Media QoL questionnaire (AOM) Dube et al. [136]	Canada	Disease-specific (Otitis Media)	Parents and children with Otitis media	English/French	10 min	Developed base on two already validated questionnaires	Four domains (sleep deprivation, change of daily and social activities, emotional distress, cancelling family plans and trips)and two domains assessing adverse consequences for the siblings and Caregiver overall QOL	13	4-point Likert and 5-point Likert	Telephone
38. Pediatric Asthma Caregivers’ Quality of Life Questionnaire (PACQLQ) Juniper et al. [137] Minard et al. [138]	Canada	Disease Specific (Asthma)	Caregivers of children with asthma	English/Spanish, Swedish, French, Portuguese, Bulgarian, Danish, Finnish, German, Chinese, Hungarian, Hebrew, Dutch, Norwegian, Persian, Polish, Russian, Serbian, Afrikaans, Arabic	3–5 min	Unstructured interviews with parents of children with asthma, a literature review and discussion with health professionals	Two domains-Activity limitations and emotional function	13	7-point Likert	Self, internet, hardcopy
39. Influenza-like illness Quality of Life (Care-ILI-QoL) Chow et al. [139]	Australia	Speciality- specific (Respiratory and infection disease)	Parents of Children With Influenza-Like Illness	English	NF	Quantitative survey, qualitative interviews with parents, and meetings with paediatricians	Four domains- Daily activities, perceived support, social life, and emotions	16	7-point Likert	Self-report
40. CAREGIVERS questionnaire Juvenile Idiopathic Arthritis (JIA) Torres-Made et al. [140]	Mexico	Disease-specific (Juvenile idiopathic arthritis)	Caregivers of children with JIA	Spanish/English	NF	Non-systematic Lit review/semi-structured interview with primary caregivers/multidisciplinary group input	Eight domains- Disease impact, social impact, economic and working impact, family impact, impact on caregiver-patient relationship, impact on couple relationship, impact on spirituality/religion/ personal beliefs, impact on social networks	28	Mixed Likert/dichotomous	Self -report
41. CD parent/caregiver QoL questionnaire (CDPC-QOL) Abreu Paiva [141]	Brazil	Disease-specific (Celiac Disease)	Parents and caregivers of Children and adolescent with Celiac disease	Brazilian-Portuguese	6 min	Developed based on Literature review, researchers experience and reviewing other QoL questionnaires	Three domains Emotions, worries, and social	30	5-point Likert	Self- report
42. Family Caregiver Quality of Life (FAMQOL) Scale [142]	USA	Disease-specific (Heart Disease -Heart Failure)	Caregivers of Heart Failure patients	English/Turkish	NF	Developed through interview with caregivers/experts	Four dimensions physical, psychological, social, and spiritual	16	5-point Likert	Self -report

**Table 2:** Psychometric properties of family quality of life measures – disease/speciality specific

Name of the measure/key references	Country	Disease/speciality	Internal consistency (Cronbach’s alpha)	Test–retest	Content	Construct/convergent	Construct/divergent/discriminant	Criterion	MID	Responsiveness/ sensitivity to change
1. Family Dermatology Life Quality Index (FDLQI) Basra et al. [92] Basra et al. [93]	UK	Specialty Specific (Dermatology)	Yes, (α = 0.88)	Yes, r = 0.94	Yes	Yes	NF	NF	NF	Yes
2. Dermatitis Family Index (DFI) L Beattie &, Lewis-Jones, [95]	UK	Disease Specific (Dermatitis)	Yes, α = 0.85 to 0.90,	Yes, (r = ·95)	Yes	Yes	NF	NF	NF	Yes
3. Parents’ Index QoL Atopic Dermatitis (PiQoL) McKenna et al. [96] Meads et al. [97]	UK, Netherlands, Germany, Italy, Spain, France, USA, Switzerland	Disease-Specific (Atopic Dermatitis)	Yes, α = 0.88 and 0.93	Yes, > 0.85	Yes	Yes	NF	NF	Yes	Yes
4. QoL in primary caregivers of children with atopic dermatitis (QPCAD) Kondo-Endo et al. [98] Katsunuma et al. [99]	Japan	Disease Specific (Atopic dermatitis)	Yes,(α = 0.66–0.87)	Yes, (r = 0.80–0.87)	Yes	Yes	NF	NF	NF	Yes
5. Childhood Atopic Dermatitis Impact Scale (CADIS) Chamlin et al. [100] Chamlin et al. [101]	USA	Disease-Specific (Atopic dermatitis)	Yes, (α = 0.76–0.93)	Yes, r = 0.96	Yes	Yes	Yes, discriminant	NF	NF	Yes
6. Psoriasis Family Index (PFI) Eghlileb et al. [102, 104]; Basra et al. [103]	UK	Disease-Specific (Psoriasis)	Yes, α = 0.86	Yes, r = 0.93	Yes	NF	NF	NF	NF	NF
7. Atopic dermatitis Burden Scale (ABS) [104]	France	Speciality- specific (Dermatology)	Yes, α = 0.78	NF	Yes	Yes	Yes, concurrent and discriminant	NF	NF	NF
8. Haemangioma Family Burden (HFB) questionnaire [105]	France	Speciality- specific (Dermatology)	Yes, α = 0.93	NF	Yes	Yes	Yes, concurrent and discriminant	NF	NF	NF
9. FamilyPso Mrowietz et al. [106]	Germany	Dermatology	Yes, α = 0.88	NF	Yes	Yes	Yes, discriminant	NF	NF	NF
10. Epidermolysis Bullosa Burden of Disease (EBBoD) Dufresne et al. [107]	France	Disease-Specific (Epidermolysis Bullosa)	Yes, α = 0.90	Yes, r = 0.97	Yes	Yes	Yes, discriminant	NF	NF	NF
11. Family Burden Ichthyosis (FBI) Dufresne et al. [108]	France	Disease-Specific (Ichthyosis)	Yes, α = 0.89	NF	Yes	Yes	Yes, discriminant	NF	NF	NF
12. Family burden of Incontentia pigmenti F’BoIP questionnaire [109]	France	Speciality-specific (Dermatology)	Yes, α = 0.93	Yes, ICC = 0·85 for each domain	Yes	Yes	Yes	NF	NF	NF
13. Parents’Diabetes QoL Questionnaire (PDQoL) Vandagriff et al. [110]; Faulkner et al. [111]	USA	Disease-Specific (Diabetes Type 1)	Yes,α = 0.64–0.9	NF	NF	NF	Yes, discriminant	NF	NF	NF
14. (WE-CARE) Cappelleri et al. [112]	USA	Disease-Specific (Diabetes Type 1)	Yes, α = 0.84–0.95	Yes, r = 0.80–0.88	Yes	Yes	Yes	Yes	NF	NF
15. Diabetes family impact scale (DFI-S) Katz et al. [113]	USA	Disease-specific (Diabetes Type 1)	Yes, α = 0.8	NF	Yes	Yes	NF	NF	NF-	NF
16. Parent Ear Nose and Throat QoL questionnaire (PAR-ENT-QoL) Berdeaux et al. [114]	France, Italy, Germany, Czech Republic, Portugal	Speciality Specific (Ear-nose-throat infection/pharyngitis)	Yes,α = 0.80–0.93	NF	Yes	Yes	Yes	NF	NF	NF
17. FAQLQ-PB Cohen et al. [115]	USA	Disease-specific (Food Allergy)	Yes, α = 0.95	Yes, r = 0.93,	Yes	Yes	Yes	Yes	NF	NF
18. Caregiver Quality of Life Cystic fibrosis (CQOLCF) Boling et al. [116]	USA	Disease-specific (Cystic fibrosis)	Yes, α = 0.91	Yes, r = 0.862,	Yes	Yes	Yes, discriminant	Yes	NF	NF
19. OverActive Bladder Family Impact Measure OAB-FIM Coyne et al. [117]	USA	Disease-specific (Overactive Gall Bladder)	Yes, α = 0.89 or greater for all sub-scales except for one 0.71	Yes, r = 0.70–0.87 ICC = 0.73 to 0.87	NF	Yes	Yes	NF	NF	NF
20. ITP-Parental burden QoL questionnaire (ITP—PB) Barnard et al. 2003 [118]	Canada, USA	Speciality Specific (Hematologic disorder)	NF	NF	Yes	Yes	NF	NF	NF	NF
21. HDQoL-C Aubeeluck and Buchanan [119]	UK	Disease-specific (Huntington’s disease)	Yes, only for sub-scales α = 0.80, 0.84, 0.89	Yes, r = 0.78, 0.86, 0.90 for Subscales	Yes	Yes	NF	NF	NF	NF
22. HDQoL-C-SF Aubeeluck et al. [120]	France, Italy	Disease-specific (Huntington’s disease)	Yes, only for sub-scales α = 0.88, 0.80	NF	NF	Yes	NF	NF	NF	NF
23. ACQL Doward, [121]	UK, France Germany, Italy, Spain	Disease-specific (Alzheimer’s)	Yes, α = 0.87 and 0.95	Yes, r = 0.93, 0.92, 0.95, 0.94, 0.90 for UK, France, Germany, Italy and Spain version	Yes	Yes	NF	NF	NF	NF
24. CAREQOL-MS Benito-Leon et al. [122]	Spain	Disease-specific (Multiple Sclerosis)	Yes, α = 0.90, 0.85, 0.81, 0.78, 0.75 for sub-scales	Yes, r = 0.96	Yes	Yes	NF	NF	NF	NF
25. PDQ-Carer Jenkinson et al. [123]	UK	Disease-specific (Parkinson’s Disease)	Yes, α = 0.92, 0.87, 0.86, 0.83 for Sub-scales	NF	Yes	Yes	NF	NF	NF	NF
26. PDQ-Carer-SI Morley et al. [124]	UK	Disease-specific (Parkinson’s Disease)	Yes, α = 0.94	NF	NF	Yes	NF	NF	NF	NF
27. PQoLCarers Pillas et al. [125]	UK	Disease-specific (Atypical Parkinsonism)	Yes, α = 0.96	NF	Yes	Yes	Yes, discriminant	NF	NF	NF
28. FOM-40 Migliorini et al. [126]	UK Australia, New Zealand, Canada	Disease-specific (Traumatic brain injury)	NF	NF	NF	NF	NF	NF	NF	NF
29. CGQOL Vickrey et al. [127]	USA	Disease-specific (Dementia)	Yes, Subscale α = 0.88, 0.93, 0.78, 0.83, 0.86, 0.86, 0.82, 0.94, 0.92, 0.89	Yes, r = 0.53–0.89	NF	Yes	NF	NF	NF	Yes
30. C-DEMQOL Brown et al. [128]	UK	Disease-specific (Dementia)	Yes, α = 0.93	NF	Yes	Yes	Yes	NF	NF	NF
31. Family Impact Scale-oro-facial disorders (FIS—OFD) Locker et al. [129]	Canada	Disease-specific (Oro-facial disorder)	Yes, α = 0.83	Yes, r = 0.80	Yes	Yes	Yes, discriminant	NF	NF	NF
32. Quality of Life in Life-Threatening Illness–Family Carer Version (QoLLTI–F) Cohen et al. [130]	Canada	Speciality Specific (Oncology)	Yes, α = 0.86	Yes, r = 0.77–0.8	Yes	Yes	NF	NF	NF	NF
33. CareGiver Oncology Quality of Life questionnaire (CarGOQoL) Minaya et al. [131]	USA	Speciality Specific (Oncology)	Yes, (0.72–0.89 except private life 0.55)	Yes, r = 0.52–0.80	Yes	Yes	Yes	NF	NF	Yes
34. Caregiver Quality of Life Index–Cancer Weitzner et al. [132]	USA	Speciality Specific (Oncology)	Yes α = 0.91	Yes, r = 0.95	Yes	Yes	Yes, divergent	Yes	NF	Yes
35. City of Hope QoL Scale–Family Version Ferrell et al. [133] City of Hope [134]	USA	Speciality Specific (Oncology)	Yes, α = 0.69	Yes, r = 0.89	NF	Factor analysis confirmed the 4 QOL domains as subscales for the instrument	NF	NF	NF	NF
36. CIQ survey Ottis Boruk et al. [135]	English	Disease-specific (Acute Otitis Media)	Yes, α = 0.88	Yes, r = 0.83,	NF	Yes	NF	NF	NF-	NF
37. Acute Otitis Media QoL questionnaire AOM-QoL) Dube et al. [136]	Canada	Disease-specific (Otitis Media)	Yes, α = 0.81	NF	Yes	Yes	Yes, discriminant	NF	NF-	NF
38. Pediatric Asthma Caregivers’ Quality of Life Questionnaire PACQLQ Juniper et al. [137]	Canada	Disease-specific (Asthma)	NF	Yes, r = 0.84	Yes	Yes	Yes, discriminant	NF	NF	Yes
39. Influenza-like illness Quality of Life Care-ILI-QoL Chow et al. [139]	Australia	Speciality Specific (Respiratory and infection disease)	Yes, α = 0.72–0.92	NF	NF	Yes	Yes, discriminant	NF	NF-	Yes
40. CAREGIVERS questionnaire JIA Torres-Made et al. [140]	Mexico	Disease-specific (JIA)	Yes, α = 0.04–0.69	Yes	Yes	Yes	Yes, divergent	NF	NF	NF
41. CD parent/caregiver QoL questionnaire (CDPC-QOL) Abreu Paiva. [141]	Brazil	Disease-specific (Celiac Disease)	Yes, α = 0.913	Yes, ICC = 0.88	Yes	NF	NF	NF	NF	NF
42. Family Caregiver Quality of Life (FAMQOL) Scale Nauser et al. [142]	USA	Disease-specific (Heart Disease-Heart Failure)	Yes,, α = 0.89	Yes, ICC = 0.91	Yes	Yes	NF	Yes	NF	NF

The review also identified Six population-specific/generic measures: their properties are summarised in Tables 3 and 4. Five of these measures (Impact on Family Scale, the Beach Centre Family Quality of Life, the PedsQL™ Family Impact Module, Family Quality of Life Survey and Care-related QoL), are aimed at specific populations of carers (parents of children, family members of people with disability, informal caregivers not necessarily family members of people with long term conditions). The only generic instrument that measures the impact of any condition on family members across all specialities is the FROM-16.

**Table 3:** Summary characteristics of family quality of life measures—population specific/generic

Name of measure/ key references	Country	Population	Language/translation	Completion time	Origin	Domains	Number of items	Scale (response options)	Mode of administration
1. PedsQL™ Family Impact Module Varni et al. [143]	USA	Parents and the family members of children with Pediatric chronic health conditions	English	NF	Developed and initially field-tested in families with medically fragile children with complex chronic medical conditions	Two domains—Parent functioning with 6 subscales measuring parents’ Self-reported functioning (physical, emotional, social, cognitive, communication worry); and family functioning with 2 subscales (daily, activities, family relationships)	36	5-point Likert	Self-report
2. Impact on-Family Scale Stein et al. [144]; Williams et al. [145]; Jalil et al. [146]	USA	Parents of children with chronic illness	English and Spanish	10 min	Family members interview	Four domains—financial, Social, personal strain and Mastery	27 (update to 15 items in 2003)	4-point Likert	Self-report, interviewer administered
3. Beach centre Family Quality of life Posten et al. [6]; Park et [147]; Hoffman et al	USA	Family members of children with disability	English, Spanish, French and Chinese	15 min	Interview with family members/focus group	Five domains-Family interaction, Parenting, Emotional Well-being, Physical/Material Well-being	25	5-point Likert	Self-report
4. Care related Quality of Life (CareQoL) Brouwer et al. [148]	Netherlands	Informal caregivers of Long term Care recipients	English/Dutch German Norwegian Swedish, Italian, Spanish and Portuguese	NF	Based on EQ-5D and evaluation of caregiver burden scales	Seven general quality of life question domains -five negative and two positive dimensions of providing informal care and VAS scale	7 and VAS question	3-point Likert	Self-report
5. Family Quality of life survey-2006, Isaac et al. [149], Perry and Isaac [150], Samuel et al. [151]	Canada	Family members of people with intellectual and developmental disabilities	English, Bosnian, Chinese, Dutch, Farsi, Flemish, French, German, Italian, Japanese, Malaysian, Polish, Romanian, Slovene, Spanish, Telugu	60 min	Expert opinion and previous research	Nine domains—health, financial well-being, family relationships, support from others, support from services, influence of values, careers, leisure and recreation, and community integration	54	5-point Likert	self-report. Interviewer administered
6. Family Reported Outcome Measure (FROM-16) Golics et al. [152]	UK	Family members of people with any health condition	English/Turkish, Thai, French and German	2 min	Qualitative interviews with family members of patients with chronic disease, Focus group and Expert panel	Two domains- Emotional, personal and social	16	3-point Likert	Self-report

**Table 4:** Psychometric properties of family quality of life measures—population specific/generic

Name of the measure/ key references	Country	Internal consistency (Cronbach’s alpha)	Test–retest	Content	Construct/convergent	Construct/divergent/discriminant	Criterion	MID	Responsiveness/sensitivity to change
1. PedsQLTM family impact module Varni et al. [143] Scarpelli [153]	USA	Yes, (α = 0.97)	Yes, r = 0.81 to 0.96	NF	Yes	NF	NF	NF	NF
2. Impact on-Family Scale (15-item) Stein et al. [144]; Jalil et al. [146]	USA	Yes, (α = 0.73)	Yes, r = 0.9	Yes	Yes	NF	NF	NF	NF
3. Beach centre Family Quality of life Posten et a. [6]; Park et al. [147]; Hoffman et al. [154]; Waschl et al. [155]; Rivard et al. [156]	USA	Yes, α = 0.88–0.94	Yes, for subscale of importance r = 0.41–0.82, for satisfaction subscale, r = 0.60–0.77	Yes	Yes	Yes, divergent and discriminant	NF	NF	Yes (French version)
4. CareQoL Brouwer et al. [148]; Hoeffman et al. [157] McCaffrey et al. [158]	Netherlands	Yes, α = 0.65	Yes, Carer 7D r = 0.55–0.94 and Carer VAS, r = 0.86	NF	Yes	Yes, discriminant	NF	NF	NF
5. Family Quality of life survey 2006 Isaac et al. [149]; Perry and Isaac [150], Samuel et al. [151]	Canada	Yes, α = 0.55–0.78	NF	Yes	Yes	NF	Yes	NF	NF
6. Family Reported Outcome Measure (FROM-16) Golics et al. [152]	UK	Yes, α = 0.80–0.89	r = 0.85–0.92	Yes	Yes	NF	Yes	NF	NF

The HRQoL instruments, regardless of having been developed for patients or their family member/partner, should demonstrate essential psychometric properties such as validity, reliability and responsiveness to change [159, 160]. Although most instruments demonstrated good internal consistency, reliability and construct validity, only 11 reported responsiveness and only one reported the MID. Thus, it is not known whether these instruments are sensitive to detecting change over time in family members' QoL.

## Discussion

This review has demonstrated that family members caring for relatives with various chronic diseases are impacted in similar ways in terms of physical, social and psychological wellbeing. The high number of FQoL instruments identified in this review demonstrates a growing interest in FQoL, though most research has focused on a few medical fields including neurology, oncology and dermatology, findings consistent with the previous review [5]. One key strength of this current review is that its findings are based on studies that have used valid tools to measure the impact of a patient's chronic disease on a family member/partner. The studies included have used many different instruments to measure the impact of chronic disease on family members, indicating a lack of consensus on the use of instruments: perhaps a clear consensus has not yet emerged because this field is still young. Furthermore, the heterogeneity of the instruments used prevents comparison of the impact of caregiving on family members across disease areas. Such comparison is important in identifying the most vulnerable family members and directing them to appropriate support. This is critical as a physically unhealthy family member would be less able to discharge their caregiving duties, thus having a negative impact on the patients' health [20]. While many studies in this review have used disease-specific instruments, most used generic health status or population instruments to measure the family impact of a person's chronic condition, indicating a strong need for a generic QoL measure specific to family members. Furthermore, most instruments used in this review have been designed keeping patients in mind and may not address issues relevant to family members. Using a measure designed to be family-specific should provide a better understanding of the needs of family members, including support services. Disease-specific FQoL instruments are used to assess QoL of family members of people with a specific disease and thus can detect changes in family member’s QoL following clinical interventions. Generic FQoL instruments on the other hand, can assess the effects of a wide range of diseases or treatment on the QoL of a partner or family member. Published research has shown that family members caring for relatives with different health conditions are impacted in similar ways [161]. Thus, generic FQoL instruments allow the comparison of QoL of individuals across different disease areas and identification of population-wide trends. While disease-specific instruments can help clinicians to understand the extent to which a partner or family member has been affected by a person’s disease and inform appropriate treatment decisions, they cannot be used to compare across conditions or between treatments. Moreover, generic instruments can measure the family impact of disease in areas where there are no disease-specific measures. Some research studies may use both generic and disease-specific instruments to capture the different patient/family member viewpoints or to validate the results of using each type of instrument. The FROM-16 could fill this gap as a generic family outcome measure since it has been developed directly from the experience of family members, for family members. One practical feature of FROM-16 is that it is a user-friendly and relatively simple questionnaire with an average completion time of 2–3 min, making it a practical tool for use in a clinical setting.

There are some limitations of this review. The review is not a systematic review. Although not a systematic review, it followed rigorous methodology and fulfilled 19 relevant PRISMA checklist items (Additional file 2: Table S1) [8]. Besides, the review only included studies in the English language, thus limiting understanding of the impact of patients' disease on family members in different cultures. Nevertheless, most studies carried out in different cultures are usually published in English language scientific journals; this suggests the amount of missed information may be minimal. Most studies in this review were cross-sectional. Only five studies were longitudinal, revealing that greater carer burden was associated with poor physical and mental health and lower QoL of family members over time, with women being impacted more than men. Future research should focus on longitudinal studies to build understanding of the long-term family impact of disease. This is important as most acute and chronic diseases may influence major life-changing decisions, thus understanding long-term impacts may help clinicians in developing better management plans for patients and their family members [162]. In addition, the majority of family members caring for relatives in the studies reviewed were women, mostly mothers. There is a dearth of research on the impact of caregiving on fathers, although this review highlighted two studies where fathers were impacted more than mothers. The fact that fathers are mostly unavailable at the point of contact results in the impact on fathers being forgotten or difficult to obtain. Thus, future research should focus on the impact of children's diseases on fathers.

An appraisal of existing FQoL instruments identified a recent plethora of FQoL measures indicating the growing recognition of the importance of FQoL. Only a few instruments have published responsiveness and MID information, however evidence of responsiveness is essential for such questionnaires to be useful for clinical monitoring or as an outcome measure to assess the value of interventions. Information concerning MID is important for the clinician to be able to interpret change in scores over time. Most instruments reviewed were developed recently, and perhaps new studies underway might later report their further psychometric properties. Further psychometric testing of existing measures is required. Furthermore, all instruments identified in this review were created in developed countries, highlighting a need for cross-cultural validation in developing countries [163].

## Conclusions

In conclusion, this review found that family members caring for their sick relative experience a huge but similar impact on their physical, social and psychological wellbeing across different disease areas. However, to translate this evidence into practice and support family members impacted by their relative's disease, there is a need for a generic family QoL measure which offers acceptable practicality and flexibility both to the relatives and to researchers as well as to clinicians. This review has identified FROM-16 as the only generic user-friendly instrument that can be implemented across all disease areas to measure the family impact of a person with a disease. However, to support the use of FROM-16 across all disciplines of medicine, there is a need for further examination of its psychometric properties. Furthermore, with greater digitalisation of healthcare, such information could be captured routinely and combined with that of the patient’s which would, no doubt, enhance the appropriateness of treatment decision-making. There are many reasons why the routine capture of quality of life information concerning patients may be helpful in enhancing the quality of clinical care [164]. Exactly similar potential advantages may be gained by the use of family quality of life measures. The final thought in this context is the utility of such instruments in meeting the aftermath challenges of the current pandemic crisis and impact of Long Covid on families of the survivors.

## Supplementary Information

**Additional file 1.** Supplementary tables-methods and results.

**Additional file 2.** Supplementary table S1-PRISMA Checklist.

## Data Availability

All data are included within the article and supplemental material.

## Abbreviations

QOL: Quality of life
FQOL: Family quality of life
HRQOL: Health-related quality of life
PRISMA: Preferred reporting items for systematic reviews and meta-analyses
ODD: Oppositional developmental disorder
FROM-16: Family reported outcome measure-16
MID: Minimal important difference
NF: Not Found
