# Correction to: Family reported outcomes, an unmet need in the management of a patient's disease: appraisal of the literature

**DOI:** 10.1186/s12955-021-01839-0

**Published:** 2021-08-31

**Authors:** R. Shah, F. M. Ali, A. Y. Finlay, M. S. Salek

**Affiliations:** 1 Division of Infection and Immunity, School of Medicine, Cardiff University, Cardiff, UK; 2 School of Life and Medical Sciences, University of Hertfordshire, Hatfield, UK; 3 Institute of Medicines Development, Cardiff, UK

## Correction to: Health Qual Life Outcomes (2021) 19:194 https://doi.org/10.1186/s12955-021-01819-4

The original article [1] contained several typos which were mistakenly incorporated by the production team that handled this article.

All errors have since been corrected.
