# Supplementary material for: Family reported outcomes, an unmet need in the management of a patient's disease: appraisal of the literature
Source: Health Qual Life Outcomes. 2021 Aug 5;19:194. doi: 10.1186/s12955-021-01819-4 (PMC8339395; doi:10.1186/s12955-021-01819-4)
Supplement: Supplementary file 1 — Additional file 1. Supplementary tables-methods and results. [file 12955_2021_1819_MOESM1_ESM.docx]

Supplemental Table S1a: Search strategy employed in OVID Medline (1946 to January 2020) to identify the articles about the impact of chronic disease on the family

| Search terms | Results |
| --- | --- |
| 1. (family or family member).mp. | 983662 |
| 2. (father or mother).mp. | 134325 |
| 3. partner.mp. | 73341 |
| 4. (parents or grandparents).mp. | 194922 |
| 5. (husband or wife or spouse).mp. | 24665 |
| 6. (Children or siblings).mp. | 1052463 |
| 7. (carer or Caregiver).mp. or Caregivers/ | 53118 |
| 8. informal caregiver.mp. | 589 |
| 9. or/1-8 | 2171560 |
| 10. quality of life .mp. | 325566 |
| 11. (impact or effect or burden or influence).mp. | 4896236 |
| 12. Secondary impact.mp. | 83 |
| 13. Family reported outcomes.mp. | 9 |
| 14. ((chronic adj disease) or (Chronic adj illness)).mp. | 293513 |
| 15. 11 or 12 or 13 | 4896243 |
| 16. 9 and 10 | 48310 |
| 17. 14 and 15 and 16 | 1251 |
| 18. limit 17 to English language | **1149** |

Supplemental Table S1b: Search strategy in OVID Medline (1946 to January 2020) extended to **include different disease areas** to capture all articles about the impact of chronic disease on the family.

| Search terms | Results |
| --- | --- |
| 1. (family or family member).mp. | 983662 |
| 2. (father or mother).mp. | 134325 |
| 3. partner.mp. | 73341 |
| 4. (parents or grandparents).mp. | 194922 |
| 5. (husband or wife or spouse).mp. | 24665 |
| 6. (children or siblings).mp. | 1052463 |
| 7. (carer or Caregiver).mp. or Caregivers/ | 53118 |
| 8. informal caregiver.mp. | 589 |
| 9. or/1-8 | 2171560 |
| 10. quality of life .mp. | 325566 |
| 11. (impact or effect or burden or influence).mp. | 4896236 |
| 12. ((chronic adj disease) or (Chronic adj illness)).mp. | 293513 |
| 13. Cardiology/ or cardiology.mp. | 49450 |
| 14. Age -related disease | 3812 |
| 15. Chronic pain.mp. or Chronic Pain/ | 43273 |
| 16. (Colorectal or Bowel disease).mp. | 8354 |
| 17. Dental disease.mp. | 1673 |
| 18. (Dermatology or Pediatric dermatology).mp. | 37320 |
| 19. (Ear, nose, and throat).mp. | 4577 |
| 20. Endocrinology.mp. or Endocrinology/ | 16706 |
| 21. Gastroenterology.mp. or Gastroenterology/ | 20789 |
| 22. General practice.mp. or General Practice/ | 47700 |
| 23. Genetic disease.mp. or Genetic disease/ | 3460898 |
| 24. Gynaecology/ or Gynaecology.mp. | 39508 |
| 25. Haematology.mp. or Haematology/ | 6421 |
| 26. Autoimmune diseases.mp. | 78520 |
| 27. Mental Health/ or Mental Health.mp. | 186996 |
| 28. Neurology.mp. or Neurology/ | 37413 |
| 29. Oncology.mp. | 115786 |
| 30. Ophthalmology.mp. or Ophthalmology/ | 38293 |
| 31. (Orthopedics or Pediatric orthopedics).mp. | 27103 |
| 32. Pediatric endocrinology.mp. | 714 |
| 33. Post Stroke.mp. | 9128 |
| 34. (Renal or renal transplant).mp. | 675972 |
| 35. Respiratory.mp. | 554008 |
| 36. Rheumatology.mp. or Rheumatology/ | 22907 |
| 37. Urology/ or Urology.mp. | 25660 |
| 38. Wound healing.mp. or Wound Healing/ | 128136 |
| 39. or/13-38 | 5415261 |
| 40. 9 and 11and 12 | 6598 |
| 41. 39 and 40 | 1786 |
| 42. 10 and 41 | 379 |
| 43. limit 42 to English language | **350** |
|  |  |

Supplemental Table S2: Search strategy* employed in OVID Medline (1946 to January 2020) to identify Existing FQoL instruments

| **Key search terms** | **Results** |
| --- | --- |
| 1. (Family* or Caregiver).mp. | 985648 |
| 2. (Quality of life or QoL).mp. | 326776 |
| 3. (Scale or Index or measure or instrument or assessment or surveys or questionnaires or inventory or tools).mp. | 3740554 |
| 4. (generic or disease specific).mp. | 70736 |
| 5. (development or psychometric or valid* or reliab*).mp. | 3543419 |
| 6. 1 and 2 | 23758 |
| 7. 3 and 4 | 23638 |
| 8. 5 and 7 | 8805 |
| 9. 6 and 8 | 257 |
| 10. limit 9 to English language | **246** |

**Search was repeated in other databases to capture all FQoL instruments*

Supplemental Table S3: Data Extraction Review studies

|  |  |  |  |  | **TYPE OF STUDY** | |  |  |  |
| --- | --- | --- | --- | --- | --- | --- | --- | --- | --- |
| **N** | **AUTHOR NAME** | **COUNTRY** | **#caregivers** | **% Female** | **CROSS-SECTIONAL** | **COHORT** | **Disease Specialty** | **Relationship to Family member** | **Tool used** |
| **1** | **Su, J. C., et al. (1997).** | Australia | 94 | NR | 1 |  | Dermatology | Parents | IOF |
| **2** | **Walsh, J. D., et al. (1999). "** | USA | 43 | 37.2 | 1 |  | Rheumatology | Parents, partner | SF36, Zarit Caregiver interview, CES-D |
| **3** | **Hunfeld, J. A., et al. (2001).** | Netherlands | 128 | 100 | 1 |  | Chronic pain | Mother | IOF |
| **4** | **Morimoto, T., et al. (2003).** | Japan | 100 | 78 | 1 |  | Neurology | Mother | SF12, Zarit caregiver interview, GDS |
| **5** | **Sewitch, M. J., et al. (2004).** | Canada | 193 | 70.5 | 1 |  | Psychiatry | Informal caregivers (Family members) | SF36, EQ-5D, GDS-15 |
| **6** | **Bruce, D. G., et al. (2005).** | Australia | 91 | 71.4 | 1 |  | Neurology | Informer caregiver | SF12 |
| **7** | **Awadalla, A. W., et al. (2006).** | Sudan | 240 | 61 | 1 |  | Endocrinology and Diabetes | Parents, wife, husband, sibling | WHOQOL |
| **8** | **Sharghi, A., et al. (2006).** | Iran | 294 | 100 | 1 |  | Haematology | Mother | BDI |
| **9** | **Blanes, L., et al. (2007).** | Brazil | 60 | 81.7 | 1 |  | Neurology | Informal caregivers (Family members) | SF36, CBS |
| **10** | **Bonner, M. J., et al. (2007).** | USA | 118 | 80.5 | 1 |  | Oncology | Mother | IOF, IES, CGSQ, PECI |
| **11** | **Forbes, A., et al. (2007** | UK | 257 | 40 |  | 1 | Neurology | Mother | SF36, CRA |
| **12** | **Gupta, V. B. (2007).** | US | 124 | 97.6 | 1 |  | Respiratory and Neurology | parents | PSI |
| **13** | **Luttik, M. L., et al. (2007).** | Netherlands | 357 | 75 | 1 |  | Cardiology | Partner | SF36, CRA, DOBI |
| **14** | **McCusker, J., et al. (2007).** | Canada | 97 | 73 |  | 1 | Psychiatry | Informal caregivers (Family members) | SF36, HAMD |
| **15** | **Arafa, M. A., et al. (2008).** | Egypt | 400 | 50.7 | 1 |  | Cardiology | Parents | SF36 |
| **16** | **Aung, L., et al. (2009).** | Singapore | 79 | 31 | 1 |  | Oncology | Parents | IOF |
| **17** | **Carod-Artal, F. J., et al. (2009).** | Brazil | 200 | 77 | 1 |  | Neurology | Informal caregivers (Family members) | EQ-5D, Zarit caregiver interview, HADS |
| **18** | **Shu, B.-C. (2009).** | Taiwan | 104 | 100 | 1 |  | Psychiatry | Mother | WHOQOL |
| **19** | **Yildirim, S. A., et al. (2009).** | Turkey | 52 | 48.1 | 1 |  | Neurology | Mother, partner, adult children | BDI |
| **20** | **Al Robaee, A. A. and M. Shahzad (2010).** | Saudia Arabia | 774 | NR | 1 |  | Dermatology | Parents | DFI |
| **21** | **Lu, L., et al. (2010)** | China | 358 | 58.1 | 1 |  | Oncology | Spouse | SF12, QoL FQ |
| **22** | **Ho, R. C. M., et al. (2010).** | Singapore | 104 | 100 | 1 |  | Dermatology | Mother | SF12, DFI |
| **23** | **Knapp, C. A., et al. (2010).** | US | 87 | NR | 1 |  | Life limiting illness | Parents | IOF |
| **24** | **Calderón, C., et al. (2011).** | Spain | 56 | 100 | 1 |  | Home enteral Nutrition | Mother | Zarit Care giver interview, |
| **25** | **Kunz, J. H., et al. (2011)** | US | 135 | 68.1 | 1 |  | Dermatology | Informal caregivers (Family members) | PedsQL 2.0 FIM |
| **26** | **Tadros, A., et al. (2011).** | Greece | 80 | NR | 1 |  | Dermatology | Informal caregivers (Family members) | FDLQI |
| **27** | **ZamZam, R., et al. (2011).** | Malaysia | 117 | 61 | 1 |  | Neurology | Informal caregivers (Family members) | WHOQOL |
| **28** | **Grant et al. (2012)** | US | 163 | 64.4 |  | 1 | Oncology | Spouse, Adult Children | CBS, IADL subscale |
| **29** | **Jirakova, A., et al. (2012).** | Czech- republic | 202 | NR | 1 |  | Dermatology | Parents | DFI |
| **30** | **Rioux, J. P., et al. (2012).** | Canada | 32 | 66 | 1 |  | Nephrology | Informal caregivers (Family member) | SF12, CBS, IES |
| **31** | **Son, K. Y., et al. (2012).** | Korean | 100 | 63 | 1 |  | Oncology | Spouse | CQOLCF |
| **32** | **Ghani, A. A. A., et al. (2013).** | Malaysia | 110 | NR | 1 |  | Dermatology | Parents | DFI |
| **33** | **Hoven, E. I., et al. (2013).** | Sweden | 551 | 73.7 | 1 |  | Oncology | Parents, Siblings | IOF |
| **34** | **Mazzone, L., et al. (2013).** | Italy | 40 | 100 | 1 |  | Internal medicine | Mother | WHOQOL |
| **35** | **Okurowska-Zawada, B., et al. (2013).** | Poland | 50 | 100 | 1 |  | Neurology | Parents | WHOQOL |
| **36** | **Haverman, L., et al. (2014).** | Netherland | 155 | 87.5 | 1 |  | Rheumatology | Parent, | (TAAQOL) |
| **37** | **Arora, A; et al .(2015)** | India | 120 | 13.3 | 1 |  | Respiratory disease, Dermatology, Psychiatry | Informal caregivers (Family member) | WHOQOL |
| **38** | **Chernyshov, P.V., et al (2015)** | multicentre-Ukraine, Czech Republic, Singapore, and Italy | 167 | NR | 1 |  | Dermatology | Mother | DFI |
| **39** | **Fleck, K., et al. (2015).** | Germany | 144 | 100 | 1 |  | Neurology | Mother | Family Impact Questionnaire |
| **40** | **Havermans, T., et al. (2015).** | Belgium | 131 | 48 | 1 |  | Oncology, Cardiology, endocrinology, Genetic diseases | Siblings | CHQ-CF 87 & SPQ. |
| **41** | **Johansson, A., et al. (2015)** | Sweden | 151 | 71.5 | 1 |  | Psychiatry | Parents/  Mother, father | SF36 |
| **42** | **Meltzer et al. (2015)** | US | 112 | 70.5 | 1 |  | Rheumatology | Parents/ mother, father | SF36 |
| **43** | **Meriggi, F., et al. (2015).** | Italy | 200 | 61.5 | 1 |  | Oncology | Informal caregiver (Family member ) | CQOLC |
| **44** | **Ridolo, E., et al. (2015).** | Italy | 90 | 26.7 | 1 |  | Allergy | Parents | Pittsburgh Sleep Quality Index |
| **45** | **AlBuhairan, F., et al. (2016)** | Saudia Arabia | 315 | NR | 1 |  | Endocrinology and Diabetes | Parents | PedsQL 2.0 FIM |
| **46** | **Chua, C. K. T., et al. (2016).** | Singapore | 16 | 56 | 1 |  | Oncology | Informal caregivers (Family members) | Zarit caregiver interview, CQOLC, CESD-R |
| **47** | **Gamwell, K. L., et al. (2016)** | US | 68 | 88 | 1 |  | Rheumatology | Mother, father | CMCRD |
| **48** | **Manee, F., et al. (2016).** | Kuwait | 71 | 100 | 1 |  | Neurology | Mother | WHOQOL |
| **49** | **Ngangana, P. C., et al. (2016).** | US | 72 | 83.3 | 1 |  | Chronic diseases / Not specified | Adult Children | Zarit caregiver interview, LSRS |
| **50** | **Nozoe, K. T., et al. (2016).** | Brazil | 32 | 100 | 1 |  | Genetic disease | Mother | Pittsburgh Sleep Quality Index |
| **51** | **Pustišek, N., et al. (2016** | Croatia | 171 | 91.2 | 1 |  | Dermatology | Parents | FDLQI |
| **52** | **Roy, A., et al. (2016)** | US | 94 | 31 | 1 |  | Gastroenterology | Partner | Zarit caregiver interview |
| **53** | **Serin, H. M., et al. (2016).** | Turkey | 100 | 100 | 1 |  | Endocrinology and diabetes, Neurology | Mother | BDI, BAI |
| **54** | **Sikorová, L. and R. Bužgová (2016)** | Czech Republic | 288 | 78.1 | 1 |  | Respiratory, Rheumatology, Endocrinology an Diabetes | Parents/  Mother, Father | PedsQL 2.0 FIM |
| **55** | **Splinter, K., et al. (2016)** | US | 32 | 54.3 | 1 |  | Genetic disease | Parents | PedsQL 2.0 FIM |
| **56** | **Van Nimwegen, K. J. M., et al. (2016).** | Netherlands | 120 | 52.2 | 1 |  | Neurology | Parents | SF12, |
| **57** | **Khair & Von Mackensen (2016).** | UK | 20 | 80 | 1 |  | Genetic disease | Parents | EQ-5D |
| **58** | **Xie, H., et al. (2016).** | China | 407 | 58.7 | 1 |  | Rheumatology , Respiratory, Neurology, cardiology , endocrinology and diabetes | Informal caregivers (Family members) | SF36 |
| **59** | **Ito, E. and E. Tadaka (2017).** | Japan | 74 | 79.7 | 1 |  | Neurology, Oncology | Informal caregivers (Family members) | CarerQoL-7D, |
| **60** | **Yilmaz et al. (2017).** | Turkey | 114 | NR | 1 |  | Respiratory | Siblings | COH QoL, PedsQLTM |
| **61** | **Guo, V. Y., et al. (2018).** | China | 255 | 100 | 1 |  | Respiratory , cardiology , Haematology, Oncology , Endocrinology Gastroenterology, internal medicine, Glaucoma | Children | CHQ-PF50 |
| **62** | **Jafari, H., et al. (2018)** | Iran | 246 | 67 | 1 |  | Respiratory, Neurology, Endocrinology Nephrology, | Informal caregivers (Family members) | WHOQOL,  Zarit caregiver burden inventory |
| **63** | **Karg, N., et al. (2018).** | Germany | 386 | 76 | 1 |  | Neurology | Informal caregivers (Family members) | CarerQoL-7D, |
| **64** | **Lynch, S. H., et al. (2018).** | USA | 168 | 72 | 1 |  | Neurology | Informal caregivers (Family members) | ProQOL |
| **65** | **Shalitin, S., et al. (2018).** | Israel | 100 | 100 | 1 |  | Endocrinology and Diabetes | Mother | WFC scale, WFF scale |
| **66** | **Wei, L., et al. (2018).** | China | 225 | 75.6 | 1 |  | Internal medicine | Informal caregivers (Family members) | Zarit caregiver interview, CQOLC-LT |
| **67** | **Al Qadire, M., et al. (2019)** | Kuwait | 264 | 78.2 | 1 |  | oncology | Parents | Zarit caregiver interview, HADS |
| **68** | **Baji, P :et al (2019)** | Hungary, Poland Slovenia | 395 | 53.1 | 1 |  | Rheumatology Respiratory Neurology Dermatology Nephrology | Informal caregivers (Family members) | EQ-5D, CarerQoL-7D, |
| **69** | **Chen, Q., et al (2019)** | USA | 54 | 71 |  | 1 | Oncology | Spouse, partner | NHP, Pittsburgh Sleep Quality Index |
| **70** | **Dinleyici, M., et al. (2019** | Turkey | 191 | 46.5 | 1 |  | Respiratory Neurology Oncology Haematology internal medicine | Siblings | PedsQL 2.0 FIM |
| **71** | **Farzi, S., et al. (2019)** | Iran | 254 | 72.4 | 1 |  | Nephrology | Informal caregivers (Family members) | SF36, Zarit caregiver interview |
| **72** | **Khair, K., et al. (2019).** | Multinational (Germany, Italy, Netherlands, Poland, Sweden, Turkey, UK) | 144 | 81.3 | 1 |  | Genetic diseases | Parents | IOF, HEMOCAB |
| **73** | **Kosan, Z., et al. (2019).** | Turkey | 90 | 36.7 | 1 |  | Genetic diseases | Informal caregiver (Family member ) | WHOQOL, Zarit caregiver interview |
| **74** | **Lazow, M. A., et al. (2019).** | US | 33 | 67 | 1 |  | Genetic diseases | Informal caregiver (Family member ) | PedsQL 2.0 FIM, CES-D |
| **75** | **Luckett, T., et al. (2019)b** | Australia | 374 | 58.6 | 1 |  | Oncology | Informal caregiver (Family member ) | SF12 |
| **76** | **Luckett, T., et al. (2019)a** | Australia | 987 | 56.7 | 1 |  | Respiratory Neurology Cardiology Oncology Psychiatry | Informal caregiver (Family member ) | SF12 |
| **77** | **Macchi, Z. A., et al. (2019** | US/Canada (Multicentre) | 175 | 73.1 | 1 |  | Neurology | Informal caregiver (Family member ) | Zarit caregiver interview, HADS |
| **78** | **Mowforth, O. D., et al. (2019)** | UK/US (Multicentre) | 53 | 60 | 1 |  | Nephrology | Informal caregiver (Family member ) | CarerQoL-7D, |
| **79** | **O’Mahony, J., et al. (2019).** | Canada | 236 | 51 | 1 |  | Neurology | Informal caregiver (Family member ) | PedsQL 2.0 FIM |
| **80** | **Suculluoglu Dikici, D., et al. (2019).** | Turkey | 68 | 45.6 | 1 |  | Neurology | Informal caregiver (Family member ) | WHOQOL |
| **81** | **Suthoff, E., et al. (2019).** | Germany, Ireland, UK,US | 88 | 85 |  | 1 | Genetic diseases | Parents | SF12, WPAI, CQOLCF, |
| **82** | **Duimering, A., et al. (2020).** | Canada | 201 | 60.8 | 1 |  | Haematology | Wife, spouse | CQOLC |
| **83** | **Farajzadeh, A., et al. (2020).** | Iran | 203 | 100 | 1 |  | Neurology | Mother | WHOQOL, BDI |
| **84** | **McDonald, L., et al. (2020).** | UK | 38 | 50.5 | 1 |  | Glaucoma | Informal caregiver (Family member ) | EQ-5D, MCSI |
| **85** | **Ten Hoopen, L. W., et al. (2020)** | Netherland | 129 | 61.2 | 1 |  | Neurology | Mother, Father | EQ-5D |
| **86** | **Wlodarek, K., et al. (2020).** | Poland | 50 | 48 | 1 |  | Dermatology | Partner | FDLQI |

**WHOQOL:** The World Health Organization Quality of Life; **SF36**: The Short Form (36) Health Survey; **SF12**:12-item Short Form Health Survey; **IOF**: Impact on Family Scale; **EQ-5D**: EuroQol- 5 Dimension; **PedsQL 2.0 FIM:** PedsQL TM 2.0 Family Impact Module; **DFI**: Dermatitis Family Impact questionnaire; **CBS:** Caregiver Burden Scale**; CarerQoL-7D**: Care-related Quality of Life instrument-7 Dimension; **BDI:** Beck Depression Inventory; **FDLQI:** Family Dermatology Life Quality Index**; CQOLC:** Caregiver Quality of Life Index-Cancer; **HADS**: Hospital Anxiety and Depression Scale; **CQOLCF:**  Caregiver Quality of Life Cystic Fibrosis**; IES:** The Impact of Event Scale; **PECI**: Parent Experience of Child Illness; **CRA**: The Caregivers Reaction Assessment Scale;  **CES-D:** Centre for Epidemiologic Studies Depression Scale; **COH-QOL:** City of Hope Quality of life Questionnaire**:**  **NHP:** The Nottingham Health profile questionnaire; **FIQ**: Family Impact Questionnaire; **PSQI:** Pittsburgh Sleep Quality Index; **PSI:** The Parenting Stress Index Questionnaire; **WPAI-SHP:** The Work Productivity and Activity Impairment-Specific Health Problem V2.0; **QoL FQ:** QoL Family Questionnaire **HAMD**: Hamilton Depression Scale; **CGSQ:** the Caregiver Strain Questionnaire. **ProQOL**: Professional Quality of Life; **GDS:** Geriatric Depression Scale; **GDS-15**: Geriatric Depression Scale-15; **CQOLC-LT:** Caregiver Quality of life index**-**Liver Transplantation; **IADL subscale:** Instrumental Activities of Daily Living; **TAAQOL:** TNO-AZL Questionnaire for Adult Health-Related Quality of life; **CHQ-CF28:** Child Health Questionnaire-Child Form-28; **SPQ:** Sibling Perception Questionnaire; **CHQ-CF87**: Child Health Questionnaire-Child Form 87; **CESD-R**: Centre for Epidemiologic Studies Depression Scale (revised); **CMCRD:** Caring for my Child with a Juvenile Rheumatic Disease; **LSRS:** Lifespan Sibling Relationship scale; **DOBI:** Dutch Objective Burden Inventory; **CHQ-PF50:** Child Health Questionnaire-Parent Form 50; **WFF:** Work-Family Facilitation scale; **WFC scale:** Work-Family Conflict scale; **PedsQLTM:** Pediatric Quality of Life Inventory TM; **HEMOCAB:** Hemophilia Associated Caregiver Burden Scale; **BAS:** Burden assessment Scale; **BAI:** Becks Anxiety Inventory; **MCSI:** Modified version of Caregiver Strain Index.

Supplemental Table S4: Number of studies and the countries where they were carried out

| **No** | **Countries** | **Number of studies** |
| --- | --- | --- |
| 1 | US | 14 |
| 2 | Turkey | 6 |
| 3 | Canada | 5 |
| 4 | Australia | 4 |
| 5 | Netherland | 5 |
| 6 | China | 4 |
| 7 | Iran | 4 |
| 8 | Brazil | 3 |
| 9 | Italy | 3 |
| 10 | Singapore | 3 |
| 11 | UK | 3 |
| 12 | Czech Republic | 2 |
| 13 | Germany | 2 |
| 14 | Japan | 2 |
| 15 | Kuwait | 2 |
| 16 | Malaysia | 2 |
| 17 | Poland | 2 |
| 18 | Saudi Arabia | 2 |
| 19 | Sweden | 2 |
| 20 | Belgium | 1 |
| 21 | Croatia | 1 |
| 22 | Egypt | 1 |
| 23 | Greece | 1 |
| 24 | India | 1 |
| 25 | Israel | 1 |
| 26 | Korean | 1 |
| 27 | Spain | 1 |
| 28 | Sudan | 1 |
| 29 | Taiwan | 1 |

| **No** | **Countries** | **Studies** |
| --- | --- | --- |
| 1 | **Hungary,** Poland, **Slovenia** (Multicentre) | 1 |
| 2 | Germany, **Ireland**, UK,US (Multinational) | 1 |
| 3 | **Ukraine**, Czech Republic, Singapore, and Italy (Multicentre) | 1 |
| 4 | Germany, Italy, Netherlands, Poland, Sweden, Turkey, UK (Multinational) | 1 |
| 5 | UK/US (Multicentre) | 1 |
| 6 | US/Canada (Multicentre) | 1 |

Supplemental Table S5: Multicentre and Multinational studies included in the review


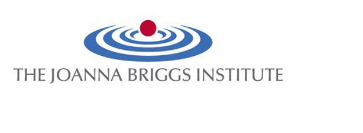
Supplemental Table S6: Joana Briggs Quality Appraisal Checklist

| JBI Critical Appraisal Checklist for Analytical Cohort Studies | | | | | | |  |  |  |  |  |  |  |  |  |  |
| --- | --- | --- | --- | --- | --- | --- | --- | --- | --- | --- | --- | --- | --- | --- | --- | --- |
| Reviewer : *Rubina Shah*  Date: June 2020 | | | | | | | Author /date | | | | | | | | | |
|  |  | | | | | | Forbes et al (2007) | | Mc Cusker (2007) | | Grant et al (2012) | | Chen et al (2019) | | Suthoff et al. (2019) | |
| 1 | Were the two groups similar and recruited from the same population? | | | | | | Yes | | Yes | | Yes | | Yes | | Yes | |
| 2 | Were the exposures measured similarly to assign people to both exposed and unexposed groups? | | | | | | Yes | | Yes | | Yes | | Yes | | Yes | |
| 3 | Was the exposure measured in a valid and reliable way? | | | | | | Yes | | Yes | | Yes | | Yes | | Yes | |
| 4 | Were confounding factors identified? | | | | | | Yes | | Yes | | Yes | | Unclear | | Yes | |
| 5 | Were strategies to deal with confounding factors stated? | | | | | | Yes | | Yes | | Yes | | Unclear | | Yes | |
| 6 | Were the groups/participants free of the outcome at the start of the study (or at the moment of exposure)? | | | | | | NA | | Yes | | NA | | NA | | Yes | |
| 7 | Were the outcomes measured in a valid and reliable way? | | | | | | Yes | | Yes | | Yes | | Yes | | Yes | |
| 8 | Was the follow up time reported and sufficient to be long enough for outcomes to occur? | | | | | | Yes | | Yes | | Yes | | Yes | | Yes | |
| 9 | Was follow up complete, and if not, were the reasons to loss to follow up described and explored? | | | | | | Yes | | Yes | | No | | NA | | Yes | |
| 10 | Were strategies to address incomplete follow up utilized? | | | | | | Yes | | Yes | | Yes | | NA | | Yes | |
| 11 | 11. Was appropriate statistical analysis used? | | | | | | Yes | | Yes | | Yes | | Yes | | Yes | |
| 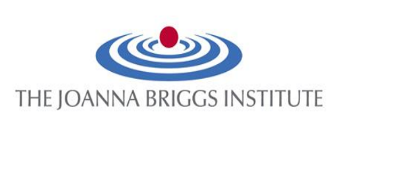  **JBI Critical Appraisal Checklist for Analytical Cross Sectional Studies** | | | | | | | | | | | | | | | |  |
| Reviewer *Rubina Shah*  Date June 2020 | | | | | | | | | | | | | | | |  |
|  | | Study author/date | Were the criteria for inclusion clearly defined? | Were the study subjects and the setting described ? | Were the exposure measured in a valid and reliable way? | Were objective, standard criteria used for measurement of the condition? | | Were Confounding factors identified? | | Were strategies to deal with confounding factors stated? | | Were the outcomes measured in a valid and reliable way? | | Were appropriate statistical analysis used? | |  |
| 1 | | Su et al. (1997) | Yes | Yes | Yes | Yes | | Yes | | Yes | | Yes | | Yes | |  |
| 2 | | Walsh et al. (1999) | Yes | Yes | Yes | Yes | | Yes | | Yes | | Yes | | Yes | |  |
| 3 | | Hunfeld et al. (2001) | Yes | Yes | Yes | Yes | | Yes | | Yes | | Yes | | Yes | |  |
| 4 | | Morimoto et al. (2003) | Yes | Yes | Yes | Yes | | Yes | | Yes | | Yes | | Yes | |  |
| 5 | | Sewitch et al (2004) | Yes | Yes | Yes | Yes | | Yes | | Yes | | Yes | | Yes | |  |
| 6 | | Bruce et al (2005) | Yes | Yes | Yes | Yes | | Yes | | Yes | | Yes | | Yes | |  |
| 7 | | Awadalla et al (2006) | Yes | Yes | Yes | Yes | | Yes | | Yes | | Yes | | Yes | |  |
| 8 | | Sharghi et al , (2006) | Yes | Yes | Yes | Yes | | Yes | | Yes | | Yes | | Yes | |  |
| 9 | | Blanes (2007) | Yes | Yes | Yes | Yes | | Yes | | Yes | | Yes | | Yes | |  |
| 10 | | Bonner et al (2007) | Yes | Yes | Yes | Yes | | Yes | | Yes | | Yes | | Yes | |  |
| 11 | | Gupta (2007) | Yes | Yes | Yes | Yes | | Yes | | Yes | | Yes | | Yes | |  |
| 12 | | Luttik et al (2007) | Yes | Yes | Yes | Yes | | Yes | | Yes | | Yes | | Yes | |  |
| 13 | | Ararafa et al (2008) | Yes | Yes | Yes | Yes | | Yes | | Yes | | Yes | | Yes | |  |
| 14 | | Aung et al (2008) | Yes | Yes | Yes | Yes | | Yes | | Yes | | Yes | | Yes | |  |
| 15 | | Carod-Artal et al (2009) | Yes | Yes | Yes | Yes | | Yes | | Yes | | Yes | | Yes | |  |
| 16 | | Shu, (2009) | Yes | Yes | Yes | Yes | | Yes | | Yes | | Yes | | Yes | |  |
| 17 | | Yildrim (2009) | Yes | Yes | Yes | Yes | | Yes | | Yes | | Yes | | Yes | |  |
| 18 | | Al Robaee, A. A. and M. Shahzad (2010). | Yes | Yes | Yes | Yes | | Yes | | Yes | | Yes | | Yes | |  |
| 19 | | Knapp, C. A., et al. (2010). | Yes | Yes | Yes | Yes | | Yes | | Yes | | Yes | | Yes | |  |
| 20 | | Ho, R. C. M., et al. (2010). | Yes | Yes | Yes | Yes | | Yes | | Yes | | Yes | | Yes | |  |
| 21 | | Lu, L., et al. (2010) | Yes | Yes | Yes | Yes | | Yes | | Yes | | Yes | | Yes | |  |
| 22 | | Calderón, C., et al. (2011). | Yes | Yes | Yes | Yes | | Yes | | Yes | | Yes | | Yes | |  |
| 23 | | Kunz, J. H., et al. (2011) | Yes | Yes | Yes | Yes | | Yes | | Yes | | Yes | | Yes | |  |
| 24 | | Tadros, A., et al. (2011). | Yes | Yes | Yes | Yes | | Yes | | Yes | | Yes | | Yes | |  |
| 25 | | ZamZam, R., et al. (2011). | Yes | Yes | Yes | Yes | | Yes | | Yes | | Yes | | Yes | |  |
| 26 | | Jirakova, A., et al. (2012). | Yes | Yes | Yes | Yes | | Yes | | Yes | | Yes | | Yes | |  |
| 27 | | Rioux, J. P., et al. (2012). | Yes | Yes | Yes | Yes | | No | | No | | Yes | | Yes | |  |
| 28 | | Son, K. Y., et al. (2012). | Yes | Yes | Yes | Yes | | Yes | | Yes | | Yes | | Yes | |  |
| 29 | | Ghani, A. A. A., et al. (2013) | Yes | Yes | Yes | Yes | | Yes | | Yes | | Yes | | Yes | |  |
| 30 | | Hoven, E. I., et al. (2013). | Yes | Yes | Yes | Yes | | Yes | | Yes | | Yes | | Yes | |  |
| 31 | | Mazzone, L., et al. (2013). | Yes | Yes | Yes | Yes | | No | | No | | Yes | | Yes | |  |
| 32 | | Okurowska-Zawada, B., et al. (2013) | Yes | Yes | Yes | Yes | | Yes | | Yes | | Yes | | Yes | |  |
| 33 | | Haverman, L., et al. (2014). | Yes | Yes | Yes | Yes | | Yes | | Yes | | Yes | | Yes | |  |
| 34 | | Chernyshov, P.V., et al (2015) | Yes | Yes | Yes | Yes | | No | | No | | Yes | | Yes | |  |
| 35 | | Fleck, K., et al. (2015) | Yes | Yes | Yes | Yes | | Yes | | Yes | | Yes | | Yes | |  |
| 36 | | Arora, A; et al .(2015) | Yes | Yes | Yes | Yes | | No | | No | | Yes | | Yes | |  |
| 37 | | Havermans, T., et al. (2015) | Yes | Yes | Yes | Yes | | Yes | | Yes | | Yes | | Yes | |  |
| 38 | | Johansson, A., et al. (2015) | Yes | Yes | Yes | Yes | | Yes | | yes | | Yes | | Yes | |  |
| 39 | | Meltzer et al. (2015) | Yes | Yes | Yes | Yes | | Yes | | Yes | | Yes | | Yes | |  |
| 40 | | Meriggi, F., et al. (2015). | Yes | Yes | Yes | Yes | | No | | No | | Yes | | Yes | |  |
| 41 | | Ridolo, E., et al. (2015). | Yes | Yes | Yes | Yes | | Yes | | No | | Yes | | Yes | |  |
| 42 | | AlBuhairan, F., et al. (2016) | Yes | Yes | Yes | Yes | | Yes | | Yes | | Yes | | Yes | |  |
| 43 | | Chua, C. K. T., et al. (2016). | Yes | Yes | Yes | Yes | | Yes | | No | | Yes | | Yes | |  |
| 44 | | Gamwell, K. L., et al. (2016) | Yes | Yes | Yes | Yes | | Yes | | Yes | | Yes | | Yes | |  |
| 45 | | Manee, F., et al. (2016). | Yes | Yes | Yes | Yes | | No | | No | | Yes | | Yes | |  |
| 46 | | Ngangana, P. C., et al. (2016). | Yes | Yes | Yes | Yes | | No | | No | | Yes | | Yes | |  |
| 47 | | Nozoe, K. T., et al. (2016). | Yes | Yes | Yes | Yes | | Yes | | Yes | | Yes | | Yes | |  |
| 48 | | Pustišek, N., et al. (2016 | Yes | Yes | Yes | Yes | | Yes | | Yes | | Yes | | Yes | |  |
| 49 | | Roy, A., et al. (2016) | Yes | Yes | Yes | Yes | | Yes | | Yes | | Yes | | Yes | |  |
| 50 | | Serin et al. (2016) | Yes | Yes | Yes | Yes | | Yes | | Yes | | Yes | | Yes | |  |
| 51 | | Sikorová, & Bužgová (2016) | Yes | Yes | Yes | Yes | | Yes | | Yes | | Yes | | Yes | |  |
| 52 | | Splinter, (2016) | Yes | Yes | Yes | Yes | | No | | No | | Yes | | Yes | |  |
| 53 | | van Nimwegen et al. (2016). | Yes | Yes | Yes | Yes | | Yes | | yes | | Yes | | Yes | |  |
| 54 | | Khair & Von Mackensen, 2016). | Yes | Yes | Yes | Yes | | Yes | | Yes | | Yes | | Yes | |  |
| 55 | | Xie, H., et al. (2016). | Yes | Yes | Yes | Yes | | Yes | | Yes | | Yes | | Yes | |  |
| 56 | | Ito, E. and E. Tadaka (2017). | Yes | Yes | Yes | Yes | | Yes | | Yes | | Yes | | Yes | |  |
| 57 | | Yilmaz, O., et al. (2017) | Yes | Yes | Yes | Yes | | Yes | | Yes | | Yes | | Yes | |  |
| 58 | | Guo, V. Y., et al. (2018). | Yes | Yes | Yes | Yes | | Yes | | Yes | | Yes | | Yes | |  |
| 59 | | Jafari et al. (2018) | Yes | Yes | Yes | Yes | | Yes | | Yes | | Yes | | Yes | |  |
| 60 | | Karg, N., et al. (2018). | Yes | Yes | Yes | Yes | | Yes | | Yes | | Yes | | Yes | |  |
| 61 | | Lynch et al 2018 | Yes | Yes | Yes | Yes | | Yes | | Yes | | Yes | | Yes | |  |
| 62 | | Shalitin, S., et al. (2018). | Yes | Yes | Yes | Yes | | Yes | | Yes | | Yes | | Yes | |  |
| 63 | | Wei, L., et al. (2018). | Yes | Yes | Yes | Yes | | Yes | | Yes | | Yes | | Yes | |  |
| 64 | | Al Qadire, et al. (2019) | Yes | Yes | Yes | Yes | | Yes | | Yes | | Yes | | Yes | |  |
| 65 | | Baji et al (2019) | Yes | Yes | Yes | Yes | | Yes | | Yes | | Yes | | Yes | |  |
| 66 | | Dinleyici et al. (2019 | Yes | Yes | Yes | Yes | | Yes | | Yes | | Yes | | Yes | |  |
| 67 | | Farzi et al. (2019) | Yes | Yes | Yes | Yes | | Yes | | Yes | | Yes | | Yes | |  |
| 68 | | Khair et al. (2019). | Yes | Yes | Yes | Yes | | No | | Unclear | | Yes | | Yes | |  |
| 69 | | Kosan et al. (2019). | Yes | Yes | Yes | Yes | | No | | No | | Yes | | Yes | |  |
| 70 | | Lazow et al. (2019). | Yes | Yes | Yes | Yes | | No | | No | | Yes | | Yes | |  |
| 71 | | Luckett(2019)b | Yes | Yes | Yes | Yes | | Yes | | Yes | | Yes | | Yes | |  |
| 72 | | Luckett et al. (2019) | Yes | Yes | Yes | Yes | | Yes | | Yes | | Yes | | Yes | |  |
| 73 | | Mowforth et al. (2019) | Yes | Yes | Yes | Yes | | Yes | | Yes | | Yes | | Yes | |  |
| 74 | | Macchi et al. (2019 | Yes | Yes | Yes | Yes | | Yes | | Yes | | Yes | | Yes | |  |
| 75 | | O’Mahony et al. (2019). | Yes | Yes | Yes | Yes | | Yes | | Yes | | Yes | | Yes | |  |
| 76 | | Suculluoglu Dikici et al. (2019). | Yes | Yes | Yes | Yes | | Yes | | Yes | | Yes | | Yes | |  |
| 77 | | Duimering et al. (2020). | Yes | Yes | Yes | Yes | | Yes | | Yes | | Yes | | Yes | |  |
| 78 | | Farajzadeh et al. (2020). | Yes | Yes | Yes | Yes | | Yes | | Yes | | Yes | | Yes | |  |
| 79 | | McDonald et al. (2020) | Yes | Yes | Yes | Yes | | Yes | | Yes | | Yes | | Yes | |  |
| 80 | | Ten Hoopen et al. (2020) | Yes | Yes | Yes | Yes | | Yes | | Yes | | Yes | | Yes | |  |
| 81 | | Wlodarek et al. (2020). | Yes | Yes | Yes | Yes | | Yes | | Yes | | Yes | | Yes | |  |
